# Superior High‐Rate Ni‐Rich Lithium Batteries Based on Fast Ion‐Desolvation and Stable Solid‐Electrolyte Interphase

**DOI:** 10.1002/advs.202413419

**Published:** 2025-02-07

**Authors:** Zhenxue Xiao, Siyuan Wu, Xiaozhe Ren, Minfei Fei, Shuai Hao, Xueping Gao, Guoran Li

**Affiliations:** ^1^ Institute of New Energy Material Chemistry School of Materials Science and Engineering Nankai University Tianjin 300350 China; ^2^ Department of Materials Science and Metallurgy University of Cambridge Cambridge CB30FS UK

**Keywords:** high‐rate performance, lithium batteries, Ni‐rich cathode, solid‐electrolyte interphase, solvation structure

## Abstract

The fast charging‐discharging performance of power batteries has very practical significance. In terms of electrochemistry, this requires fast and stable kinetics for electrochemical reaction processes. Despite the great complexity of kinetics, it is clear that lithium‐ion desolvation and a subsequent step of crossing through cathode‐electrolyte interphase (CEI) are crucial to high‐rate performance, in which the two key steps depend heavily on the working electrolyte formula. In this work, a customized electrolyte is developed to coordinate ion desolvation and interphase formation by introducing vinylene carbonate (VC), triphenylboroxin (TPBX), and fluoroethylene carbonate (FEC) but excluding ethylene carbonate (EC). Serving Ni‐rich cathodes, the customized electrolyte generates a double‐layered CEI, LiF‐dominated inorganics inner layer, and ROCOOLi‐dominated organics outer layer, which is not only stable and very efficient for lithium ion transport. Meanwhile, a ${\mathrm{PF}}_{6}^{-}$‐dominated solvation structure is induced and effectively decreases the desolvation energy to 29.72 kJ mol^−1^, supporting fast lithium ion transport in the cathode interfacial processes. Consequently, the Ni‐rich lithium‐ion battery achieves a stable long cycle at a superior high rate of 10 C.

## Graphical abstract

A customized electrolyte for coordinating ion desolvation and interphase formation of superior high‐rate Ni‐rich lithium batteries is developed. A stable double‐layered CEI is generated, and a ${\mathrm{PF}}_{6}^{-}$‐dominated solvation structure is induced to effectively decrease the desolvation energy, both of which support fast lithium ion transport in the cathode interfacial processes.

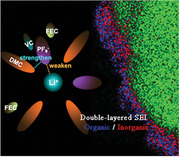

## 1 Introduction

With incredible speed, electric vehicles powered by lithium‐ion batteries (LIBs) have penetrated into the daily lives of people. LIBs with high‐energy density are a technical basis of long mileage for electric vehicles. That is why Ni‐rich cathode materials such as LiNi_0.8_Co_0.1_Mn_0.1_O_2_ (NCM811) with high capacity are increasingly used in commercial power LIBs.^[^ ^1^ ^]^ However, the unsatisfactory performance of vehicle batteries at high rates is actually limiting further development of electric vehicles, because poor rate performance means low output power and long charging time. Especially, for the emerging electric low‐height aircraft, enough high rate performance of power batteries is necessary. To meet this challenge, some studies focus on improving the transportation speed of lithium ions inside the electrode materials, including shorting ion transport distance by reducing particle sizes,^[^ ^2^ ^]^ widening ion channels by doping metal ions,^[^ ^3^ ^]^ decreasing ion transfer energy barriers by surface coating^[^ ^4^ ^]^ and so on. These strategies are effective to some extent, but not enough for achieving a high enough rate performance because, beyond the cathode material itself, the electrolytes play a decisive role in it. The electrolyte is the carrier of the lithium‐ion transfer between electrodes, and the rate capability of ion transfer seriously determines the fast charging and discharging ability of the cell. At present, the researches on high‐rate electrolytes of LIBs are mainly focused on improving the ionic conductivity of electrolytes, increasing the migration number of lithium ions, generating stable interfacial layers, and constructing weak solvation structures.^[^ ^1^, ^5^ ^]^ In a word, the realization of high rate electrolyte is to improve the transfer kinetics of lithium ions from the two perspectives of the bulk electrolyte and the electrode electrolyte interphase, and the interfacial process is considered to be the rate determining step of the electrochemical reaction during the fast charging of the cell.^[^ ^6^ ^]^ The transfer of lithium ions at the electrode electrolyte interphase is divided into two steps, namely, the desolvation of lithium ions and the subsequent crossing of the interfacial layer. It is generally believed that the weak binding between lithium ions and solvents is conducive to the desolvation of lithium ions at the interphase.^[^ ^7^ ^]^ Meanwhile, the inorganic rich interfacial layer has high ionic diffusivity and can inhibit the thickening of the interfacial layer caused by continuous corrosion reaction between electrolyte and electrode.^[^ ^8^ ^]^ Therefore, it is effective to construct a lithium‐ion solvation structure with weak Li^+^‐solvent interaction and a tendency to generate an inorganic rich interfacial layer for achieving excellent high rate performance.

The solvation structures of lithium ions are a competitive result of coordination between lithium ions with different solvents and salt anions of electrolytes, which depend on the different ratios of solvent‐separated ion pairs (SSIPs), contact ion pairs (CIPs), and aggregates (AGGs) in the solvation sheath.^[^ ^8^, ^9^ ^]^ The different solvation structures spontaneously have different desolvation energy barriers. This influences not only the desolvation step in kinetics but also subsequently components of solid‐electrolyte interphase (SEI) which is another crucial factor for the performance of batteries and includes CEI and anode‐electrolyte interphase (AEI). It has been found that adjusting the solvent‐derived organic‐rich phase into anion‐derived inorganic‐rich phase could make the cathodes and the lithium deposition more stable.^[^ ^10^ ^]^ Even, the low‐temperature performance could be enhanced by changing SEI.^[^ ^11^ ^]^

To regulate the solvation structure of lithium ions in electrolytes, the easiest approach is to change the rational compositions of the mixed solvent.^[^ ^12^ ^]^ However, the difficulty lies in finding a delicate balance between the desolvation process and SEI composition. By partially replacing the solvents in traditional carbonate electrolytes with weakly solvating analogues, more anions in salts can take part in CIPs and AGGs to form inorganic‐dominated CEI.^[^ ^13^ ^]^ Following this direction, high concentrations of electrolytes (over 5 M) were early used to influence solvation structure.^[^ ^14^ ^]^ Afterward, localized high‐concentration electrolytes were developed by adding diluent at the outer layer of solvation structures.^[^ ^15^ ^]^ In the typical electrolytes for lithium‐ion batteries, EC solvent has a strong solvation effect and is involved in the solvation sheath of lithium ions, leading to organic‐rich interphase layers with high resistance, low mechanical strength, and easy breaking.^[^ ^16^ ^]^ More seriously, it can be catalyzed by highly oxidized transition metal ions in Ni‐rich cathodes to cause a dehydrogenation reaction.^[^ ^17^ ^]^ It also obstacles the desolvation of lithium ions and severely limits the rate performance.^[^ ^16a^ ^]^ Therefore, in order to improve NCM's rate performance further, the EC should be avoided.^[^ ^18^ ^]^ With this consideration, the electrolyte needs to be further regulated with EC excluded. Meanwhile, the viscosity, voltage window, as well as ionic conductivity should be maintained at a suitable level.

In this study, a customized electrolyte is developed for commercial NCM811 cathodes. EC is removed to eliminate its adverse side reaction with NCM; and FEC, VC, and TPBX are added to enable fast lithium‐ion transportation during the desolvation process until crossing the interphases. Under a super‐high rate of 10 C, NCM811 can cycle stably for 800 cycles. To reveal the electrochemical mechanism in this electrolyte, especially at the interphase with NCM811, a series of advanced characterization methods together with molecular dynamics (MD) calculations are utilized to characterize the CEI and solvation structure. Moreover, the self‐discharging tests, high‐voltage tests, and pouch cell tests are systematically carried out to verify its comprehensive performance further.

## 2 Results and Discussion

### 2.1 Electrolyte Compositions and Properties

**Figure 1:**
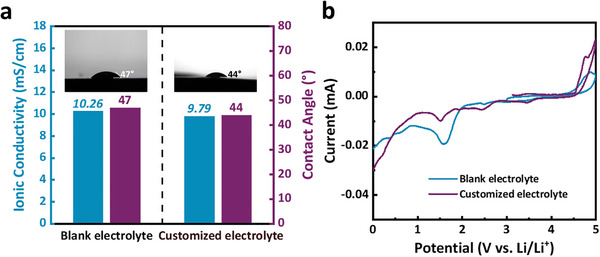
a) Ionic conductivities and contact angles of the electrolytes at RT. b) Cyclic voltammetry curves of the electrolytes with a scanning speed of 0.1 mV s^−1^ and an open circuit voltage of 2.8 V.

EC solvent is purposely avoided in consideration of its adverse reaction with NCMs. However, the EC absence can decrease the dissociation degree of lithium salt LiPF_6_, as reflected in the reduced ionic conductivity in Figure S1 (Supporting Information). Thus, to keep ionic conductivity, the LiPF_6_ concentration is increased to 1.5 m, and with that comes a rising viscosity that is not desirable for the wettability of electrolytes on electrodes and separators (Figure S2, Supporting Information). Therefore, a fine trade‐off among these parameters has to be made through a series of tests. In addition, the oxidative or reduction decompositions of additives (TPBX, VC, and FEC) in preference to EC are utilized to build stable interfacial layers at both the cathode side and anode side (The binding energies and energy levels of the solvents and additives with lithium ions are shown in Figure S3, Supporting Information). Based on lots of investigations as described in the Experiment section, the optimized electrolyte formula is 1.5 M LiPF_6_ in DMC, with 4 vol% VC, 1 wt% TPBX, and 6 vol% FEC as additives (Figure S4, Supporting Information). This electrolyte is marked as a customized electrolyte (CE). For comparison, a typical recipe, 1 M LiPF_6_ dissolved in EC: DMC = 3:7, is employed as a blank electrolyte (BE). As shown in **Figure** **1**a, the ionic conductivity and contact angle of the customized electrolyte are comparable to that of the blank electrolyte. Though TPBX causes an oxidation peak at 4.9 V, the customized electrolyte shows a wider potential window between 0.5 and 4.7 V in comparison with the blank electrolyte, which is suitable for Ni‐rich cathode material (Figures 1b and S5, Supporting Information). Moreover, the customized electrolyte has smaller reduction peaks at a low potential above 0.5 V, meaning less electrolyte consumption on the anode side.

### 2.2 Electrochemical Performance

**Figure 2:**
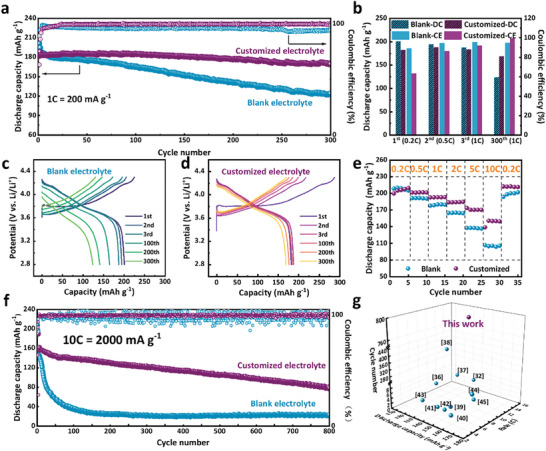
a) Cycle performance at room temperature with 1 C rate. b) The changes in discharge capacity and Coulombic efficiency in the initial three cycles and the 300th cycle. c,d) Charge‐discharge curves for the specific numbers of cycles. e) Rate performance in two electrolytes. f) Long cycle performance with a 10 C rate at room temperature. g) High‐rate performance data from the published works.

The performance of the customized electrolyte is evaluated by the capacity and cycling stability of NCM811 cathodes. Galvanostatic cycling at 1 C rate shows that NCM811 in the customized electrolyte maintains a specific discharge capacity of 169 mAh g^−1^ after 300 cycles, achieving a capacity retention of 92.05% (**Figure** **2**a; Figure S6, Supporting Information). For comparison, only 123.8 mAh g^−1^ with a capacity retention of 66.10% can be obtained in the blank electrolyte. Moreover, the average Coulombic efficiency in the customized electrolyte reaches 99.61% after electrochemical activation in the initial cycle, much higher than 95.79% in the blank electrolyte. The detailed changes in discharge capacity and Coulombic efficiency reveal there exists an electrochemical activation process during the initial few cycles in the case of the customized electrolyte, and the Coulombic efficiency keeps remarkably increasing (Figure 2b). The activation process reflects the formation of CEI and AEI layers different from the typical one by complex electrochemical reactions. Obviously, NCM811 in the customized electrolyte deliveries a whopping initial charge capacity of 288.1 mAh g^−1^, because new interphases are generated by involving the additives in the electrolyte (Figure 2c,d). During this process, FEC can decompose at ≈3.4 V, as proven by the CV results in Figure S7 (Supporting Information). Directly, this leads to the low initial Coulombic efficiency.^[^ ^19^ ^]^ Notably, the charge and discharge medium voltages of the Ni‐rich cathode are much more stable in the customized electrolyte than in the blank electrolyte, as shown in Figure S8 (Supporting Information). It clearly indicates that electrochemical polarization is significantly reduced when the new interphases form. This point is actually very important for vehicle batteries, because stable voltage not only maintains high energy density but also facilitates the management of the power systems.^[^ ^20^ ^]^ Generally, the customized electrolyte provides better performance in discharge capacity, cycling stability, and voltage stability.

As mentioned above, the customized electrolyte is designed to support a superior high rate performance. It can be found that, the higher the rate is, the more remarkable the advantage in rate performance of the customized electrolyte is (Figure 2e). Even at a super‐high rate of 10 C, the discharge capacity is up to 150 mAh g^−1^. Moreover, when the current density returns to 0.2 C, the discharge capacity can completely recover. Considering the urgent requirement for fast charging/discharging in practical applications of electric vehicles, a long cycling test under a large current density of 2000 mA g^−1^, i.e., 10 C rate, also meaning charging/discharge within 6 min, is carried out. As shown in Figures 2f and S9 (Supporting Information), the customized electrolyte supports NCM811 to deliver an initial discharge capacity of 160 and 78 mAh g^−1^ is remained after cycling for 800 times at 10 C rate. To our knowledge, the cycle stability under such a high rate breaks through the previous records (Figure 2g).^[^ ^21^ ^]^ For the blank electrolyte, the initial discharge capacity of NCM811 is 146 mAh g^−1^ and drops sharply to a measly 35 mAh g^−1^ after only 100 cycles due to the fast degradation of NCM811 under such a high current density. Meanwhile, the Coulombic efficiency in the customized electrolyte is also much more stable with an average value of 99.5%, compared to 98.2% with erratic fluctuation in the blank electrolyte. The performance under a super‐high rate of 10 C makes ultra‐fast charging/discharging in electrical vehicles available. It also shows the great importance of electrolyte regulation and its huge influence on improving the dynamic process of lithium‐ion batteries.

### 2.3 Electrochemical Kinetics

**Figure 3:**
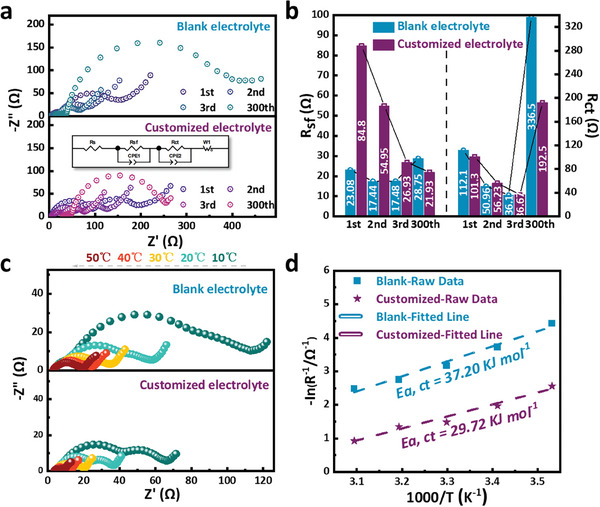
a) EIS spectra of NCM811 after different cycles at 1C rate. b) The fitted R_sf_ and R_ct_ and their changes after different cycles, corresponding to a). c) Temperature‐dependent EIS spectra of electrochemically activated NCM811 electrodes in the electrolytes. d) Arrhenius behavior of the R_ct_ in c), corresponding to the lithium ions desolvation process.

Such a stable high‐rate performance points to stable and fast electrochemical kinetics, especially at the interphases where there are usually high energy barriers for charge transport. Electrochemical impedance spectroscopy (EIS) of the fully charged cells after the 1st, 2nd, 3rd, and 300th cycles are shown in **Figure** **3**a. Their interfacial behaviors can be characterized by surface resistance R_sf_ and interface charge transfer resistance R_ct_. The former represents the impedance of lithium ions going through the SEI layer, and the latter implies the difficulty of charge transfer during the interphase reaction (which is also known as the electrode polarization impedance).^[^ ^22^ ^]^ Both of the two resistances gradually decrease during the initial three cycles corresponding to the electrochemical activation stage. The trend is regardless of electrolyte compositions, reflecting the process from formation to stabilization of CEI and AEI (Figure 3b). However, the value of R_sf_ for the customized electrolyte is larger than that for the blank electrolyte, again suggesting a more complex interfacial reaction and different interphase formation. As the cycling continues, R_sf_ decreases obviously and is lower than the one in the blank electrolyte, indicating that the interphases form finally and keep stable in the initial few cycles, and there is continuous electrolyte decomposition and interphase growth. As for R_ct_, it has close values in the two electrolytes at the activation stage. However, a large difference appears after 300 cycles, 192.5 ohm for the customized electrolyte and 336.5 ohm for the blank electrolyte. This suggests an important understanding that CEI and AEI layers in the customized electrolyte are much more favorable for charge transfer at the electrochemical interphases. Conclusively, in the customized electrolyte, distinct and beneficial interphases between electrode and electrolyte are generated to support outstanding electrochemical performance.

More profound informations about electrochemical kinetics come from temperature‐dependent EIS. As shown in Figure 3c, the impedance in the case of the customized electrolyte is much lower than that in the blank electrolyte in all the tested temperatures. The results prove strongly that the customized electrolyte is fully qualified to work in a wide range of temperatures with little temperature dependence. The R_ct_ values at various temperatures are fitted according to the Arrhenius law, and the corresponding energy barriers of lithium‐ion desolvation are obtained.^[^ ^7^, ^23^ ^]^ As shown in Figure 3d, the activation energies of the desolvation process in the customized electrolyte and the blank one are 29.72 and 37.20 kJ mol^−1^, respectively. The values reflect the highest energy barriers in the complete kinetic process. Here they reveal that the desolvation of lithium ions in the customized electrolyte is easier than that in the blank one. This supports the fast charging/discharging ability under the super‐high rate of NCM811 in the customized electrolyte shown in Figure 2f. This ratiocination will be further confirmed afterward. Therefore, these EIS results indicate, from a view of electrochemical kinetics, that the customized electrolyte induces the changes of two important issues: one is the interphase constitution between electrodes and electrolyte, and the other is the desolvation difficulty degree of lithium ions.

### 2.4 Structure and Composition of Cathode‐Electrolyte Interphase

**Figure 4:**
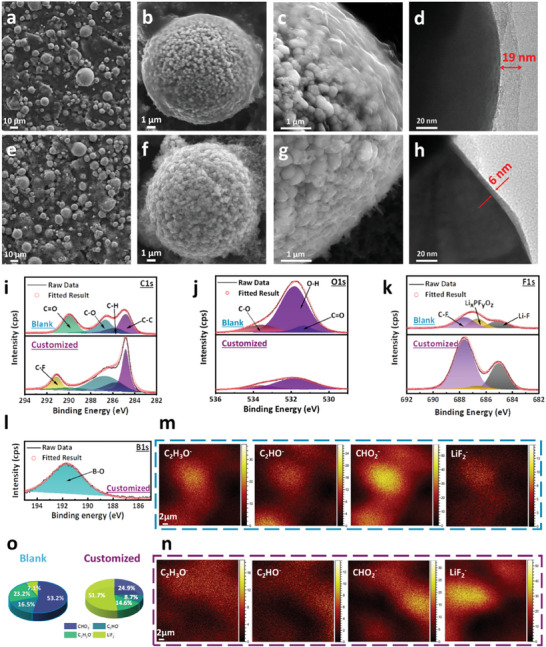
a–c) SEM and d)TEM images of the cycled NCM811 particles in the blank electrolyte. e–g) SEM and h) TEM images of the cycled NCM811 particles in the customized electrolyte. i‐l) X‐ray photoelectron spectroscopy of CEI layers in the electrolytes. TOF‐SIMS mappings of CEI layers in the (m) blank electrolyte and (n) the customized electrolyte. o) The percentages of surface species content in CEI layers in the electrolytes, as derived from TOF‐SIMS results.

To investigate the structure and composition of CEI, the batteries with the customized and the blank electrolyte are disassembled after 300 cycles to analyze the Ni‐rich cathodes in detail. Generally, the NCM811 sphere‐like particles with a diameter of 5–25 microns, assembled by primary particles with a size of 200–500 nm, are completely kept in morphology, and no cracks are found after the long cycling (**Figure** **4**a,b,e,f). On the surface of the secondary particles, a layer of film can be observed at high magnification, which is easily recognizable compared to a fresh NCM811 (Figure 4c,g; Figure S10a,b, Supporting Information). There is no obvious difference between the cycled cathode materials in the customized and the blank electrolyte on the level of the electrode and even secondary particles. However, when the surface of the primary particles is focused on TEM observation, a clear difference appears, i.e., a thin amorphous interphase with a thickness of ≈6 nm in the customized electrolyte, while ≈19 nm in the blank electrolyte (Figure 4d,h; Figure S10c, Supporting Information). Surface sensitive XPS characterizes the composition of the interphases. The relative intensity ratio of the carbon's deconvoluted peaks, between C─O (286.6 eV), C─H (285.7 eV), and C═O (289.8 eV) peaks to C─C (284.8 eV) and C─F (291.1 eV) peaks, is obviously lower from the customized electrolyte than from the blank (Figure 4i). This means that organic components such as ROCOOLi are dominant in the interphase formed in the blank electrolyte, whereas inorganic interphase such as LiF are mainly generated in customized electrolyte. The organics come from the decomposition of the electrolyte solvents and cover the signal of the conductive agent and the binder. Consistent with the results of C1s, the fitted peaks of C─O (533.6 eV) and C═O (531.3 eV)^[^ ^24^ ^]^ from the O1s spectra (Figure 4j) are also lower in the case of the customized electrolyte. In the F1s spectra (Figure 4k), the Li_x_PF_y_O_z_ (686.6 eV) peaks are notably reduced in the customized electrolyte, indicating that the adverse decomposition of lithium salt is obviously restrained due to the absence of EC in the customized electrolyte.^[^ ^17b^ ^]^ Li‐F peak is significantly higher in the CEI in the customized electrolyte, which should come from the decomposition of FEC, as the decomposition mechanism can be inferred from Scheme S1 (Supporting Information).^[^ ^19^ ^]^ In addition, the presence of a B─O peak in Figure 4l confirms the involvement of TPBX in the interphase. The reaction mechanism can be described (Scheme S2, Supporting Information): the ring‐opening polymerization of TPBX generates oligomers with alternating B─O chains, then promotes the transfer of lithium ions.^[^ ^21k^ ^]^ As a consequence, different from the organic‐rich CEI in the blank electrolyte, more inorganic species containing F and B are formed in the CEI in the customized electrolyte.

Based on the above results, it can be concluded that the customized electrolyte actually changes the composition of the interphase on the surface of NCM811, and inorganics dominate the interphase. As well known, the composition, structure, thickness, and properties of the interphase can decisively influence mass transfer kinetics in electrochemistry. Furthermore, the chemical composition distribution and proportion are visualized by Time‐of‐Flight Secondary Ion Mass Spectrometry (TOF‐SIMS), and Figure 4m,n display the species fragments tested on the surface of cycled NCM811elctrodes in the two electrolytes. In comparison, there are much stronger signals of LiF_2_^−^ and much weaker signals of CHO_2_^−^, C_2_HO^−^, and C_2_H_3_O^−^ in the case of the customized electrolyte. A normalization calculation demonstrates that the CEI in the blank electrolyte contains more than 90% organic phase, while the one in the customized electrolyte is composed of over 50% inorganics, like LiF_2_^−^ (Figure 4O). Combined with the XPS and SIMS results, it is clear that the inorganics are mainly LiF. Usually, the inorganics with higher mechanical strength can avoid the problem of repeating rupture and generation in organic CEI layers.

**Figure 5:**
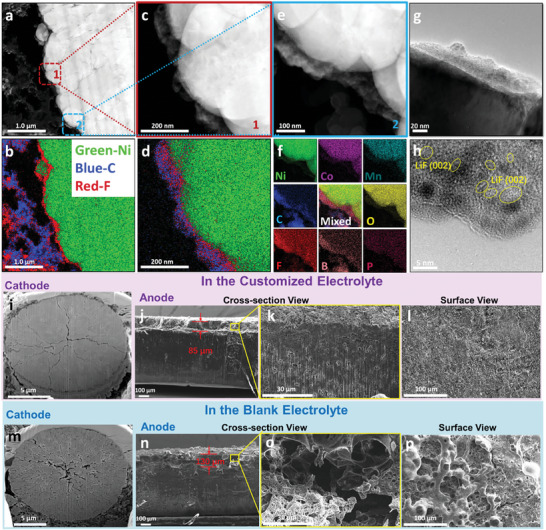
a–f) TEM images and corresponding EDS mapping images of CEI layers (red‐F, blue‐C, green‐Ni). g,h) HRTEM images of CEI layers. i–p) FIB‐SEM of the cycled NCM811 particles in (i–l) the customized electrolyte and (m‐p) the blank electrolyte.

Another important question worth probing is whether there is any unique structure for the inorganics and organics in the CEI layer to support the long‐term stability available. In **Figure** **5**a, a quarter of the NCM811 secondary particle is imaged in a thin sheet with nanometer primary particles resolved via FIB‐TEM technology. Its EDS mapping clearly shows that the F element distributes richly along the edge of the NCM811 particle (Figure 5b). As shown in the two enlarged magnification images (Figure 5c,e), the CEI layer is clearly observed on the surface of the particle due to its different contrast. Their EDS mappings confirm that the CEI layers comprise mainly C and F elements, corresponding to the organic and inorganic phases, respectively (Figure 5d,f). Notably and interestingly, the inorganic and organic compositions exhibit a unique structure: LiF sticks closely to the particle surface as the inner layer of CEI, and the organics are located at the outer layer. The total thickness of the CEI layer is between 30–80 nm for the sample cycling 800 times at a 10 C rate, thicker than that cycled at 1C rate. This indicates a fast formation process of CEI layers under a superior‐high current density, enabling the protection function at a high rate. It can be observed from the bright field image of HRTEM in Figure 5g,h that the CEI layer is composed of grainy regions. The lattice fringes are widely observed with a lattice spacing of 0.212 nm and assigned to the (200) plane of the LiF. The double‐layered interphase structure is representative, and such a structure can be observed on the random NCM811 particles (Figure S11, Supporting Information). The unique structure of CEI plays a very important role in achieving stable and fast mass transfer kinetics. On the one hand, the inorganic LiF in the inner layer with high mechanical strength combines the organic phase in the outer layer with high flexibility can inhibit fractures and meanwhile accommodate the strains correspondingly. On the other hand, the electronic insulation of LiF prevents the continuous growth of CEI layers, and the thin CEI layer is conducive to the rapid transport of lithium ions.^[^ ^25^ ^]^

The outcome of the double‐layered CEI is reflected in alleviating the crack of NCM811 particles. As compared in Figure 5i,m, NCM811 particles cycled in the blank electrolyte generate some wide cracks from its center and spread radially along numerous grain boundaries. However, in the customized electrolyte, the NCM811 particles have much fewer gracile cracks, and the primary particles keep close contact with each other. It has been reported that one of the prime reasons for the performance deterioration of NCM cathode is the expanding cracks originating from the center of the material particles, which is caused by the strain difference between the layered phase and the electrochemically inactive phase and the columbic repulsion generated by the Ni‐Li anti‐site defect.^[^ ^26^ ^]^ Thanks to the high mechanical strength of double‐layered CEI, it can withstand the stress between particles to a large extent during charge and discharge, thereby reducing the generation of stress‐induced cracks. In addition, the uniform double‐layered CEI on the surface of the secondary particles can effectively inhibit the electrolyte from entering the already generated internal microcracks, so as to avoid the formation of a large amount of CEI at the crack and crack intensification. For sure, CEI layers cannot completely and intrinsically adjust the internal stress, but the alleviation effect is noteworthy.

Certainly, for a long and stable cycling of batteries, the anode side is equally important, especially for AEI. Viewed from both the surface and the cross‐section of the lithium anode, as shown in Figure 5j–l,n–p, the lithium deposition is quite dense and uniform in the customized electrolyte, but very sparse and uneven in the blank electrolyte. It is beneficial from the F‐rich AEI at the upper surface of deposition layers, as convinced in the previous works^[^ ^8^, ^11^ ^]^ and is also observed in the EDS mapping in Figure S12 (Supporting Information) in this work. Therefore, the customized electrolyte can still induce effective AEI layers on the anode side with EC absence and meanwhile benefit the CEI layer.

### 2.5 Solvation Structure

**Figure 6:**
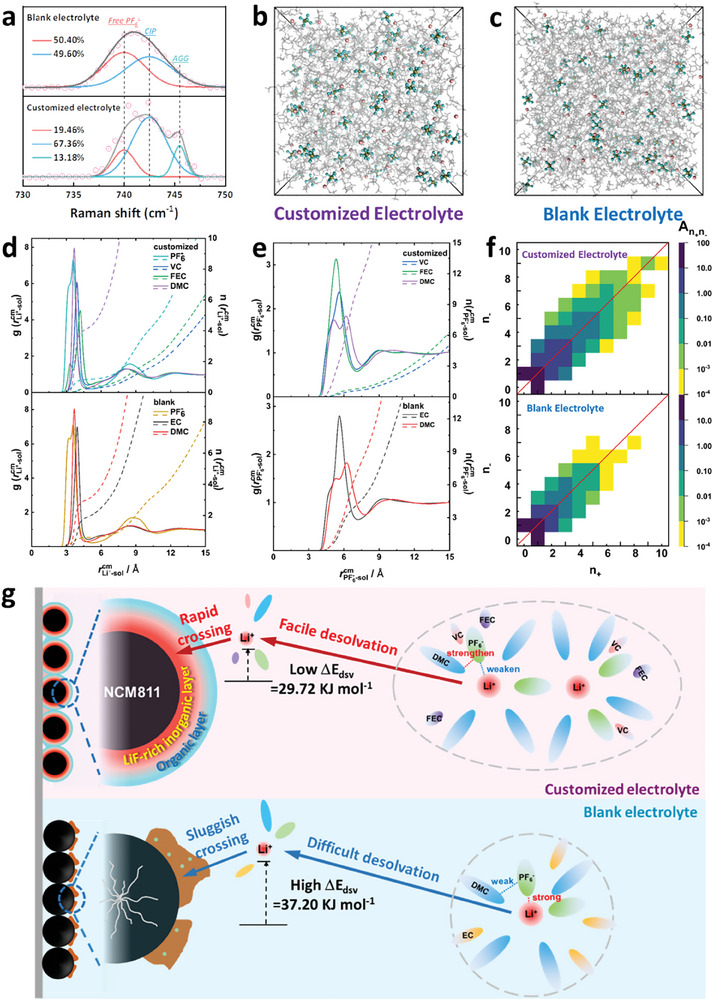
a) Raman spectra of the customized and the blank electrolyte. Snapshots of b) the customized and c) the blank one taken from MD simulations, in which Li (pink), P (yellow), and F (green) are shown in ball and stick, and the solvent molecules are shown in wireframes. d) Atomistic site‐site RDFs (solid lines), $g(r_{\mathrm{Li}-\mathrm{sol}}^{ss})$’s, between lithium ions and the solvents and P‐atom in ${\mathrm{PF}}_{6}^{-}$, and the corresponding CDFs $n(r_{\mathrm{Li}-\mathrm{sol}}^{ss})$’s (dashed lines). e) Center‐of‐mass RDFs (solid lines), $g(r_{{\mathrm{PF}}_{6}^{-}-\mathrm{sol}}^{cm})$’s, between ${\mathrm{PF}}_{6}^{-}$ and solvent molecule sol, with the corresponding CDFs $n(r_{{\mathrm{PF}}_{6}^{-}-\mathrm{sol}}^{cm})$’s (dashed lines). f) Distribution of the average number of A_ij_, made of i = n Li^+^and j = m ${\mathrm{PF}}_{6}^{-}$. g) Schematic illustration of function mechanism.

The formation of CEI has been widely confirmed to be closely related to the composition of the electrolyte solvation sheath on the electrode surface.^[^ ^8^, ^9^, ^13^ ^]^ Thereupon, the solvation structures of two electrolytes are first studied by Raman spectra (Figure S13, Supporting Information). The vibrational spectra of anion speciation are quite different in the two electrolytes in the frequency range of 730–750 cm^−1^, as shown in **Figure** **6**a.^[^ ^12^, ^15^, ^27^ ^]^ After fitting to three different Voigt (Gauss‐Lorentz) functions, the peaks centered at ≈740, 742.5 and 745.5 cm^−1^ are attributed to free PF_6_^−^, CIPs and AGGs, respectively. The CIPs and AGGs represent an anion interacting with one Li ion and two or even more Li ions, respectively.^[^ ^16^, ^28^ ^]^ In the blank electrolyte, only free PF_6_^−^ and CIP exist and are essentially evenly split, so the solvated lithium ions are largely shielded by solvents. In contrast, in the customized electrolyte, free PF_6_^−^ and CIPs account for 19.46% and 67.36% respectively, and AGGs is 13.18%. It means that the CEI and AEI will be more derived from the decomposition of LiPF_6_, increasing the content of inorganics (like LiF) in it.^[^ ^8^ ^]^ More anions in the solvation structure cause lower solvation energy and lead to rapid desolvation processes.^[^ ^9^, ^29^ ^]^ It shows the customized electrolyte is a weakly solvating electrolyte,^[^ ^13^, ^23^ ^]^ which can induce the generation of an anion‐dominated interphase between the electrode and electrolyte and achieve rapid lithium‐ion desolvation.^[^ ^11a^ ^]^

In detail, Figure 6b,c shows randomly selected snapshots in the blank and the customized electrolytes. Two types of CIP are found between Li^+^ and ${\mathrm{PF}}_{6}^{-}$: that is the monodentate CIP with Li^+^ binding to a single F‐atom in ${\mathrm{PF}}_{6}^{-}$, and bidentate CIP with Li^+^ binding to two F‐atom in ${\mathrm{PF}}_{6}^{-}$.^[^ ^30^ ^]^ As shown in the RDFs of $g(r_{\mathrm{Li}-\mathrm{sol}}^{\mathrm{cm}})$’s in Figure 6d, the shoulder peak of $\;g(r_{\mathrm{Li}-{\mathrm{PF}}_{6}^{-}}^{\mathrm{cm}})$’s below 3 Å in the first solvation shell is slightly depressed in the customized electrolyte, while the main peak ≈3.5 Å is enhanced, as compared to those in the blank electrolyte. It indicates that the bidentate CIP is depressed and the monodentate CIP is enhanced with the presence of FEC and VC in the customized electrolyte. Since the binding between Li^+^ and ${\mathrm{PF}}_{6}^{-}$ in the bidentate CIP is stronger than that in the monodentate CIP, it is expected that the dissociation of lithium ions is easier in the customized electrolyte.

In the RDFs of $g(r_{{\mathrm{PF}}_{6}^{-}-\mathrm{sol}}^{cm})$ in Figure 6e, $g(r_{{\mathrm{PF}}_{6}^{-}-\mathrm{DMC}}^{\mathrm{cm}})$ with the peak ≈5.2 Å in the customized system is sharper and slightly shifted to closer contact as compared to that in the blank system. These indicate a stronger solvation effect on ${\mathrm{PF}}_{6}^{-}$ in the customized system than that in the blank system. The strong ${\mathrm{PF}}_{6}^{-}$‐DMC interaction can suppress the reduction decomposition on the cathode surface and promote the formation of anion derived CEI layer.^[^ ^31^ ^]^ In brief, as illustrated in Figure 6f, the ${\mathrm{PF}}_{6}^{-}$ in the customized electrolyte is and strongly solvated by the solvent in it, then it is binding with lithium ions is weakened, leading to an easier desolvation process for lithium ions and facilitating the formation of an inorganic rich interfacial layer.

Besides, the coordination numbers of lithium ions with the oxygens in DMC are increased from 2.86 in the blank electrolyte to 3.9 in the customized electrolyte. It refers to a larger amount of AGGs. This is also reflected in the distribution of molecular clusters illustrated in Figure 6f. Once there are more AGGs in the electrolyte, a large number of salt anions will accumulate on the electrode surface and be preferentially decomposed, forming an anion‐derived CEI layer rich in inorganic phase, so as to achieve uniform transmission of lithium ions with fast kineties (Figure 6g).^[^ ^9^, ^12^ ^]^

### 2.6 Practical Application for Batteries

**Figure 7:**
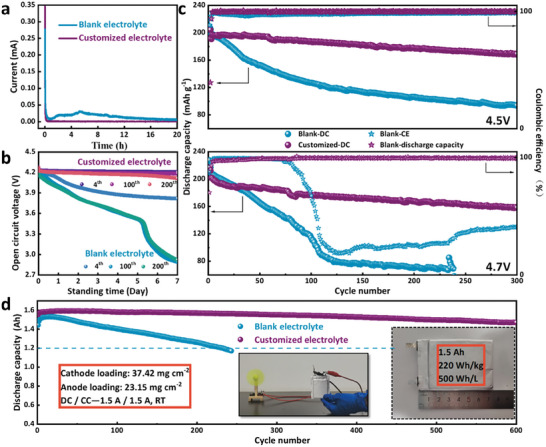
a) Leakage currents of Li/NCM811 cells after electrochemical activation. b) Self‐discharge curves of Li/NCM811 cells standing for 7 days after different cycling numbers. c) Cycling performance of NCM811 electrodes with charge cut‐off voltage on 4.5 and 4.7 V at 1 C. d) Long cycling performance of 1.5 Ah Graphite/NCM811 pouch cells with a current of 1.5 A.

In view of the practical application, fast and stable kinetics are not enough for an outstanding battery. First, the self‐discharge test is used to evaluate battery performance in storage at a static state.^[^ ^32^ ^]^ The leakage current is recorded at a fully charged state after the electrochemical activation of NCM811. As shown in **Figure** **7**a, it is much smaller and more stable in the customized electrolyte compared to the blank one. The changes of OCV are monitored when the Li/NCM811 cells are set aside at a fully charged state for 7 days, and the conditions were compared after different cycling times, including 4, 100, and 200 cycles. In the customized electrolyte (Figure 7b), the OCV drops within 7 days are only 0.07, 0.09, and 0.15 V after 4, 100, and 200 cycles, respectively. The situation drastically worsens in the blank electrolyte. It drops 0.43 V after the 4th cycle, and 1.3 V after the 100th cycle with all power lost (Figure S14a, Supporting Information). After the three times 7‐day intervals, the cells continued cycling to test their capacity decay (Figure S14b, Supporting Information). There is 95.21% capacity retention in customized electrolyte after the subsequent 300 cycles. However, in blank electrolyte, only 19.85% capacity is remained under the same conditions. One of the main inducement of self‐discharge behavior is the side reaction between electrode and electrolyte, which causes the change of electrode potential. Therefore, the robust and stable interfacial layer formed in customized electrolyte can significantly improve the self‐discharge behavior of NCM by inhibiting the harmful side reactions.

With EC excluded, the NCM811 in the customized electrolyte can be charged to a higher voltage at 4.5 and 4.7 V (Figure 7c), which meets the need for higher energy density. The cells perform stably for 300 cycles with capacity retention of 85.74% (168.9 mAh g^−1^, 4.5 V) and 78.58% (158.1 mAh g^−1^, 4.7 V). In contrast, the capacity in the blank electrolyte drops to merely 93.6 mAh g^−1^ and 41.4 mAh g^−1^. The voltage curves and CV curves in the two electrolytes are compared in Figure S15 (Supporting Information).

In a very practical way, the customized electrolyte is verified in 1.5 Ah pouch cells of graphite/NCM811. It exhibits good long‐cycling ability with 94.48% capacity retention after 600 cycles (Figure 7d). In the blank electrolyte, the capacity quickly decays to 80% retention after only 250 cycles. A smaller polarization is observed in the customized electrolyte through the voltage curves and median voltage in Figure S16 (Supporting Information). The integrity of both NCM811 and graphite particles is well reserved in the postmortem SEM images (Figure S17, Supporting Information).

## 3 Conclusion

In summary, a new electrolyte with VC, FEC, and TPBX as additives and EC excluded is specially developed for NCM811‐based lithium‐ion batteries. The customized electrolyte significantly supports the corresponding batteries to achieve superior high‐rate performance, working stably for 800 cycles at 10 C. In mechanism, this is attributed to fast and stable electrochemical kinetics in the customized electrolyte, which is realized by influencing the two key issues, CEI reconstruction and ion desolvation acceleration. The double‐layered CEI, LiF‐rich inorganics inner layer, and organics outer layer are stable and very efficient for ion transport. And a ${\mathrm{PF}}_{6}^{-}$‐dominant solvation structure of lithium ions decreases desolvation energy to 29.72 from 37.20 kJ mol^−1^ in the customized electrolyte. Moreover, the customized electrolyte shows minimized self‐discharge and stable cycling ability in the high cutoff voltage (4.7 V) in very practical measurements.

## 4 Experimental Section

### Preparation of Electrolytes

The blank electrolyte was 1 M LiPF_6_ in EC and DMC (volume ratio of 3:7), purchased from Nanjing MJS Energy Technology Co., LTD., China. The other studied electrolytes mentioned in this paper were all configured by ourselves, and the specific formulas were shown in Table S1 (Supporting Information). All of the reagents were purchased from Shanghai Titan Technology Co., LTD., China, and all of the electrolytes were prepared and stored in an argon‐filled glovebox (Kejing, oxygen <0.1 ppm, water <0.1 ppm) at room temperature.

### Preparation of 2032 coin cells

The electrodes were prepared by mixing 80 wt % LiNi_0.8_Co_0.1_Mn_0.1_O_2_ (Tianjin Lishen Battery Joint‐Stock Co., LTD., China), 10 wt % Super P, and 10 wt % polyvinylidene fluoride (PVDF) in N‐methylpyrrolidone (NMP), coating the slurry on Al foil. After vacuum drying at 110 °C for 12 h, the electrodes were cut into slices with a diameter of 1 cm and surface loading of 2 mg cm^−2^. Li/LiNi_0.8_Co_0.1_Mn_0.1_O_2_ 2032 type coin cells were assembled with Celgard 2400 as a separator in the argon‐filled glovebox.

### Preparation of 1.5 Ah pouch cells

The cathode electrodes were composed of 97.35 wt.% LiNi_0.8_Co_0.1_Mn_0.1_O_2_, 0.75 wt.% CNT, 0.50 wt.% SP, and 1.40 wt.% PVDF, and the areal density was 37.42 mg cm^−2^. The anode electrodes were composed of 95.70 wt.% LiNi_0.8_Co_0.1_Mn_0.1_O_2_, 1 wt.% SP, 1.30 wt.% CMC, and 2 wt.% SBR, and the areal density was 23.15 mg cm^−2^. All of the electrodes were provided by Tianjin EV Energies Co., Ltd., China. Aluminum and nickel strips were attached as electrode tabs to the sides of the cathode and anode, respectively. The electrolyte addition for each pouch cell was 5 g Ah^−1^. The energy densities can reach 220 Wh kg^−1^ and 500 Wh L^−1^.

### Electrochemical Measurements

Galvanostatic cycling of Li/LiNi_0.8_Co_0.1_Mn_0.1_O_2_ coin cells was conducted on a CT2001A LAND test system (1C = 200 mA g^−1^). The cyclic performance of pouch cells was tested on a LANHE test system (CT6001A), which was charged at a constant current of 1.5 A to 4.2 V, then at a constant voltage to a current of less than 75 mA, and finally discharged at a current of 1.5 A and repeated. Electrochemical impedance spectroscopy was carried out on the IM6ex electrochemical station (Autolab, Germany) in a frequency range of 10^5^ −10^−2^ Hz with a potential amplitude of 5 mV. Linear scanning voltammetry (LSV) and cyclic voltammograms (CV) were performed on an LK2010 electrochemical workstation with a scan rate of 0.1 mV s^−1^. All of the above tests were conducted at room temperature.

### Material Characterizations

The ionic conductivities of electrolytes were measured using a DDS‐11A (YOKE INSTRUMENT) with a Pt electrode at 25 °C. The contact angles of electrolytes were measured with a JC2000D3M measuring instrument. The solvation structure of electrolytes was characterized on a Micro‐confocal Raman spectrometer (SR‐500I‐A) with a laser wavelength of 785 nm. Morphology and structure tests were performed on FE‐SEM (JSM‐7800F) at 15.0 kV and TEM (JEM‐2800F). X‐ray photoelectron spectroscopy (XPS) measurements were performed on a Thermo Scientific ESCALAB 250Xi. The time‐of‐flight secondary ion mass spectrometry (TOF‐SIMS) adopted the negative ion mode uniformly, and the 2D scanning area was 100 × 100 µm^2^.

## Conflict of Interest

The authors declare no conflict of interest.

## Author Contributions

G.L. conceived the project. Z.X. prepared and tested the samples. S. W. and X. R. performed the theoretical calculations. S. H. measured the TEM and FIB parts and analyzed the results. M. F. and X. G. analyzed the electrochemical data. G.L. supervised the entire project. Z.X. and S.H. wrote the first draft of the paper. G. L. rewritten and reviewed the final version. All authors discussed the results and contributed to the writing of the paper.

## Supporting information



Supporting Information

## Data Availability

The data that support the findings of this study are available from the corresponding author upon reasonable request.
