# Supplementary material for: Superior High‐Rate Ni‐Rich Lithium Batteries Based on Fast Ion‐Desolvation and Stable Solid‐Electrolyte Interphase
Source: Adv Sci (Weinh). 2025 Feb 7;12(12):2413419. doi: 10.1002/advs.202413419 (PMC11948075; doi:10.1002/advs.202413419)
Supplement: Supplementary file 1 — Supporting Information [file ADVS-12-2413419-s001.docx]

Supporting Information

**Superior High-Rate Ni-Rich Lithium Batteries Based on Fast Ion-Desolvation and Stable Solid-Electrolyte Interphase**

*Zhenxue Xiao, Siyuan Wu, Xiaozhe Ren, Minfei Fei, Shuai Hao^*^, Xueping Gao, and Guoran Li^*^*

**Electrolyte design and regulation**

One role of EC in electrolytes is to participate in the formation of SEI to passivate the anode electrode surface, which reduces the continuous decomposition of electrolytes. From the LSV curves in Figure S5a, it can be seen that for the electrolyte with only 1.5 M LiPF_6_ in DMC, there is a strong reduction peak at 1.7 V, which means that the removal of EC from the electrolyte will inevitably lead to the anode electrode surface being unable to be passivated, resulting in a large amount of decomposition of the electrolyte. There is no doubt that the discharge capacity of the Li/NCM811 cell assembled with this electrolyte also rapidly decays and cannot be used normally, as shown in Figure S4a. VC, as one of the most successful anode film-forming additives so far, can effectively solve the above problem in small amount. From Figure S5a and S4b, it can be found that a trace amount of VC can greatly inhibit the reduction decomposition of electrolyte at low potential, and significantly improve the cycle stability of the cell, of which 4 vol% concentration has the best effect. This is because VC can participate in the formation of the anode SEI layer, passivating the anode surface, and avoiding the continuous rapid decomposition of the electrolyte on the anode surface.

**Figure S1.** Ionic conductivities and contact angles of different electrolytes at RT.

**
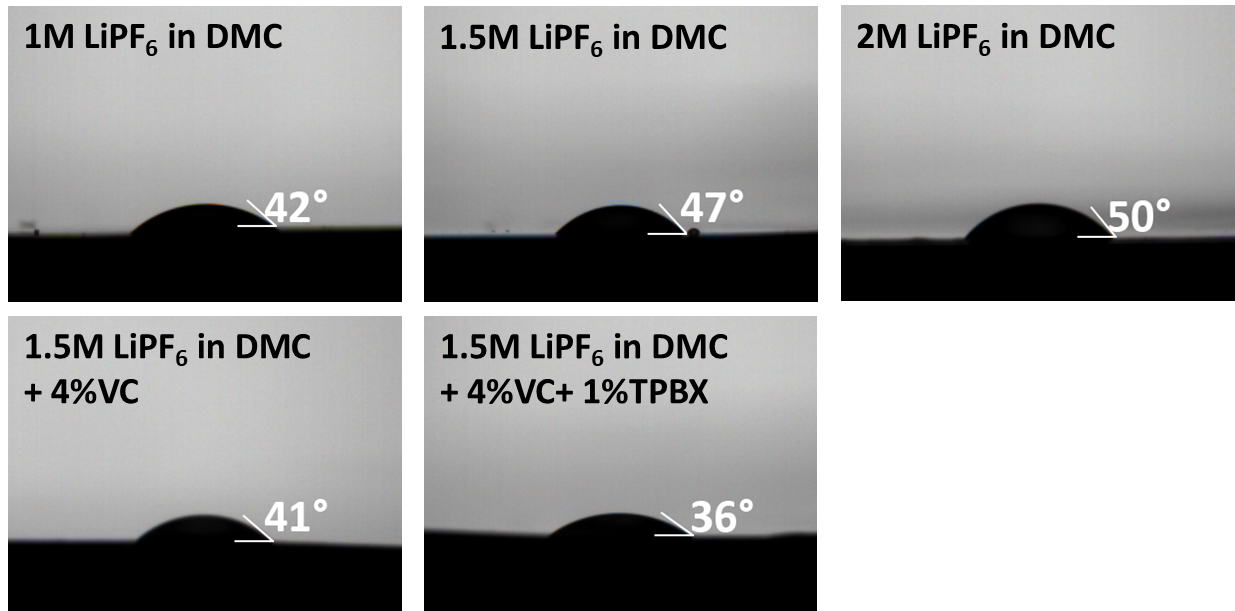
**

**
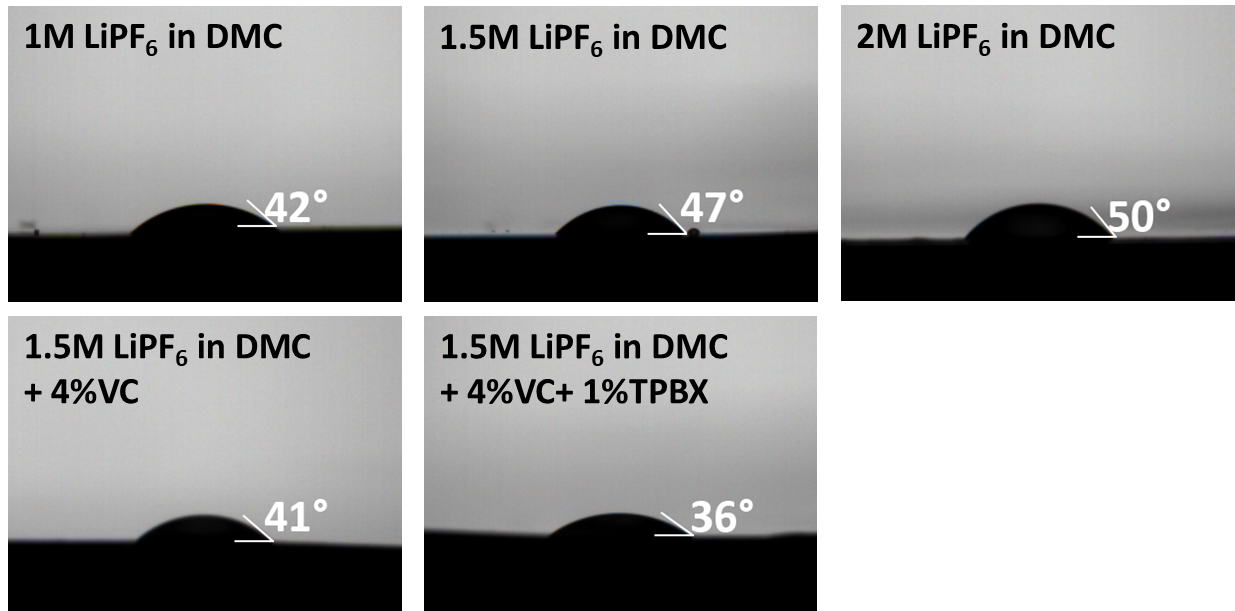
**

**Figure S2.** Contact angle optical photographs of different electrolytes at RT.


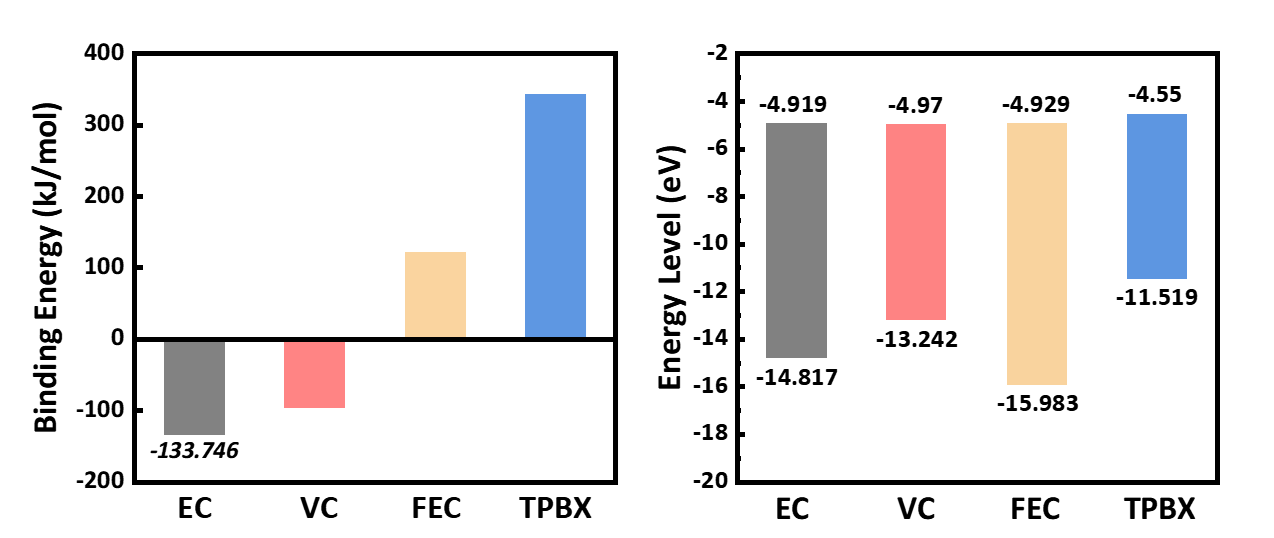


**Figure S3.** Binding energy and energy level of Li-EC, VC, FEC and TPBX.

**
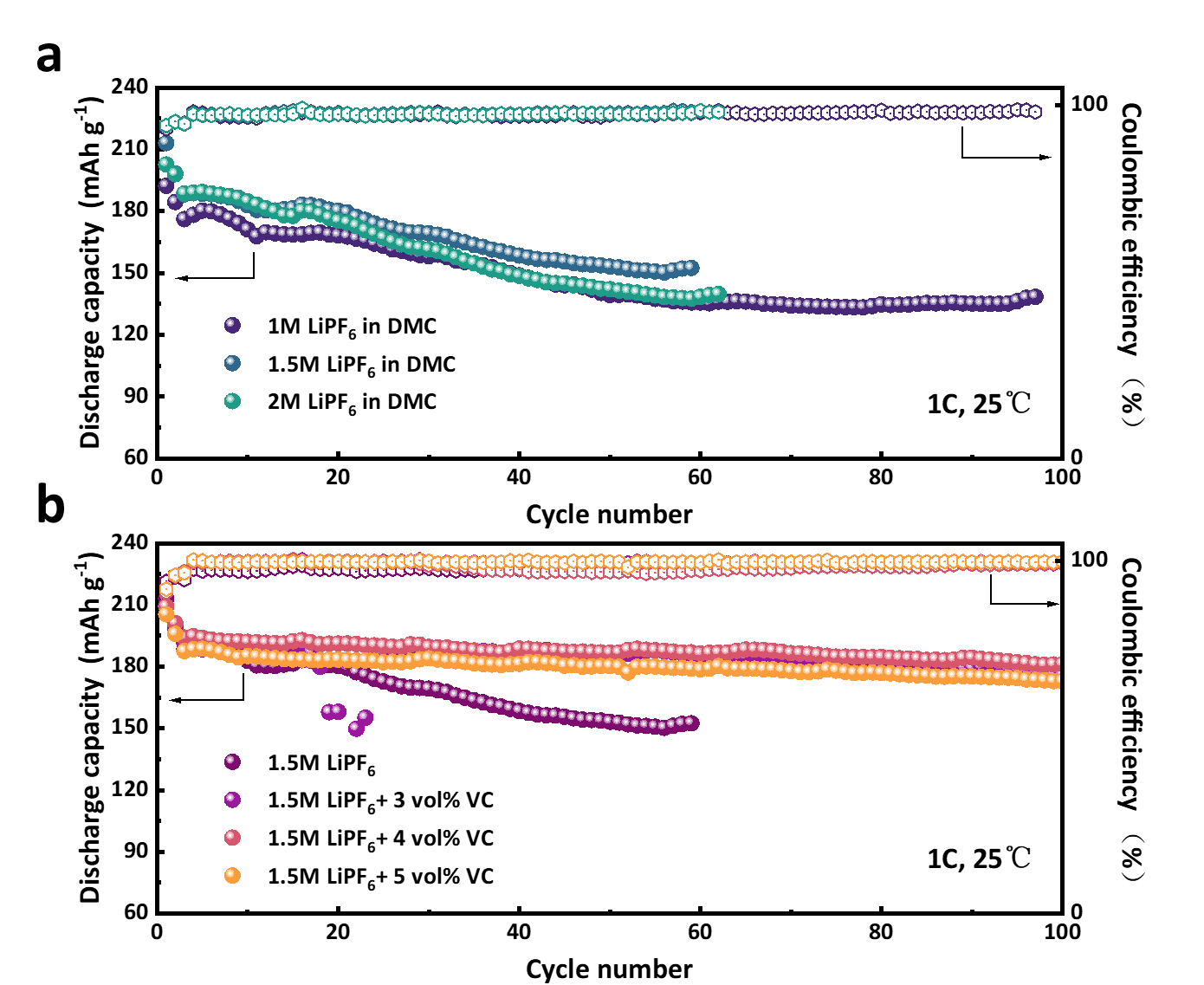
**

**Figure S4.** Cycling performance of Li/NCM811 cells in electrolytes with different a) LiPF_6_ concentrations and b) VC concentrations.

As shown in Figure S4a, the discharge capacity of the Li/NCM811 cell assembled with LiPF_6_ in DMC electrolyte rapidly decays and cannot be used as normal. VC, as one of the most successful anode film-forming additives so far, can effectively solve the above problem in small amounts. From Figure S4b and S5a, it can be found that a trace amount of VC can greatly inhibit the reduction decomposition of electrolyte at low potential, and significantly improve the cycle stability of the cell, of which 4 vol% concentration has the best effect. This is because VC can participate in the formation of the anode SEI layer, passivating the anode surface, and avoiding the continuous rapid decomposition of the electrolyte on the anode surface.

**
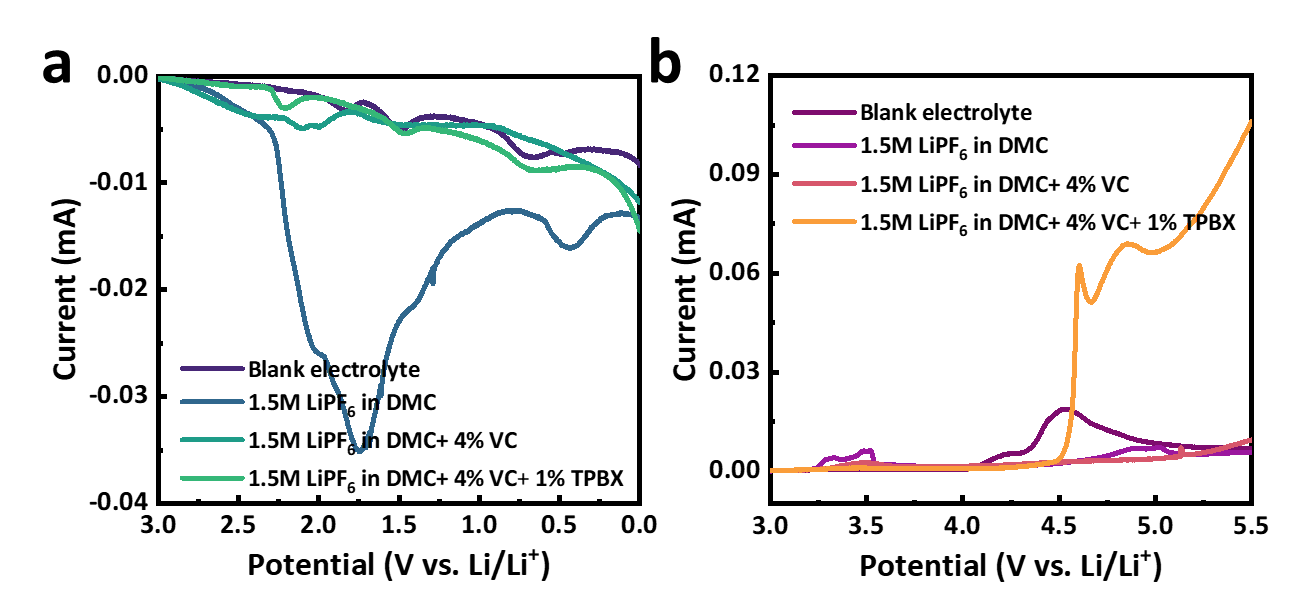
**

**Figure S5.** LSV curves of different electrolytes with a scanning speed of 0.1 mV s^-1^ towards a) low potential and b) high potential.

Compared with the LSV curve of 1.5 M LiPF_6_ in DMC with 4 vol% VC and 1 wt% TPBX electrolyte in Figure S5b, the customized electrolyte in Figure 1c will have a weak oxidation peak when the potential is higher than 3.4 V, which indicates that FEC will undergo oxidation decomposition under this potential. More notably, by comparing the electrolyte before and after adding TPBX in Figure S5b, it is found that after the addition of TPBX, there will be a strong oxidation peak when the potential is higher than 4.6 V, which represents a large amount of decomposition of TPBX. In summary, compared to the blank electrolyte, the customized electrolyte has lower reduction decomposition at low potential, whereas FEC and TPPBX will decompose by oxidation at specific potential.


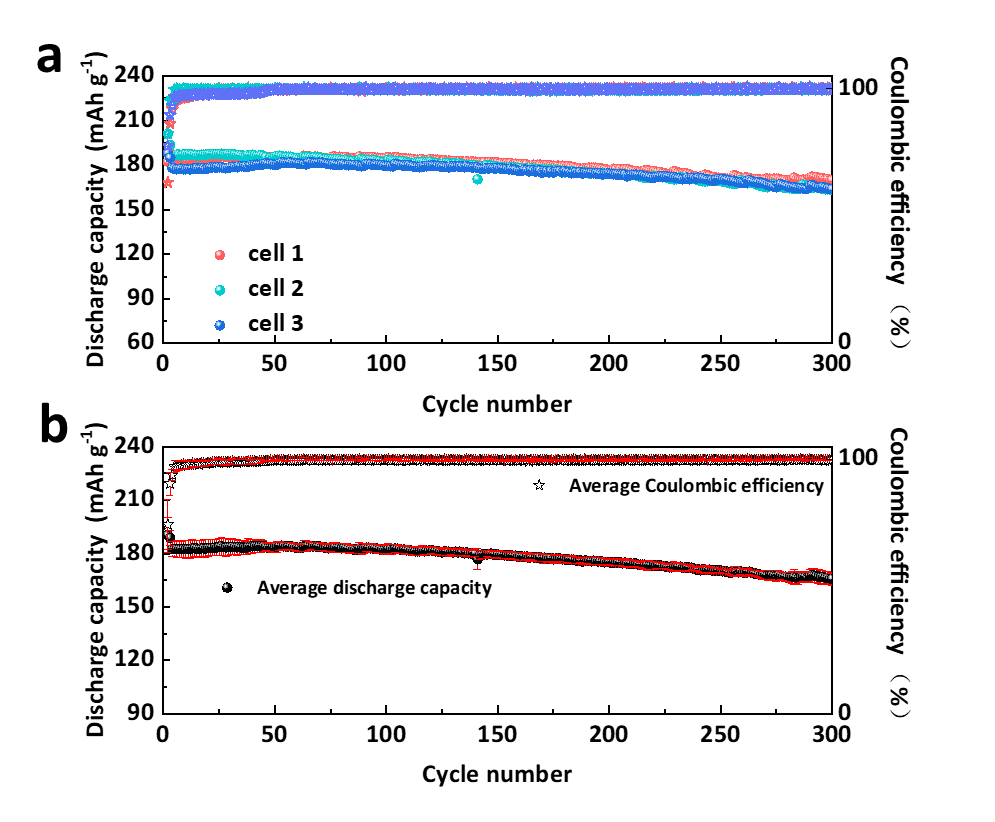


**Figure S6.** a) Cycling performance of three Li/NCM811 cells under the same conditions with customized electrolyte and b) their average data at 1C rate.


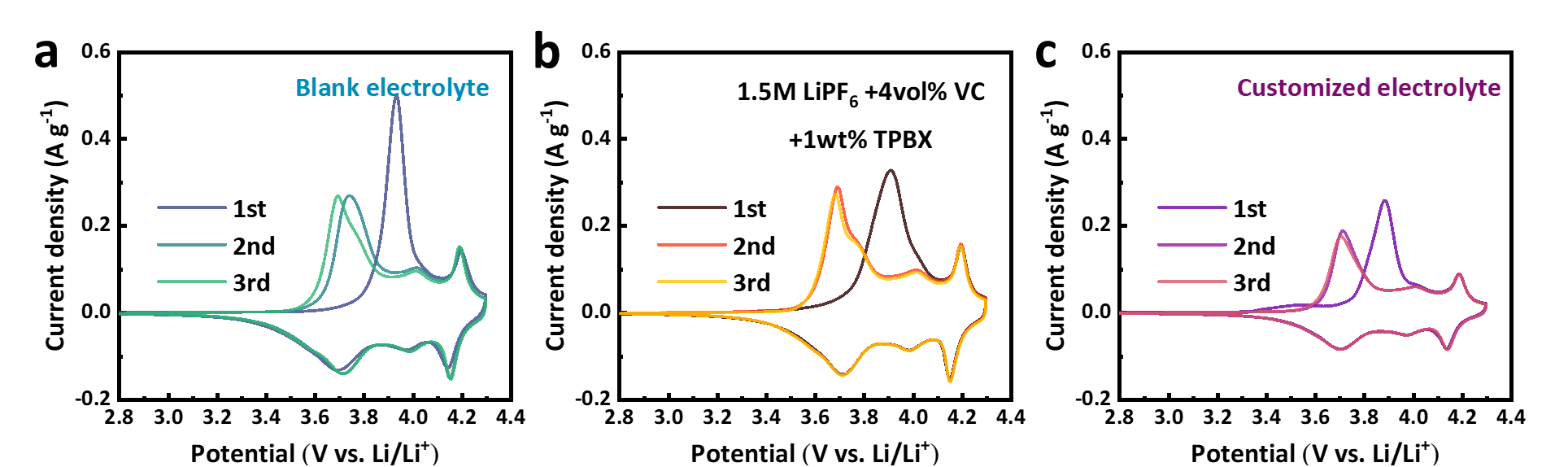


**Figure S7.** a-c) CV curves of NCM811 electrodes in different electrolytes with a scanning speed of 0.1 mV s^-1^.

**Figure S8.** Charge and discharge medium voltage of NCM811 electrodes during 1C cycling.


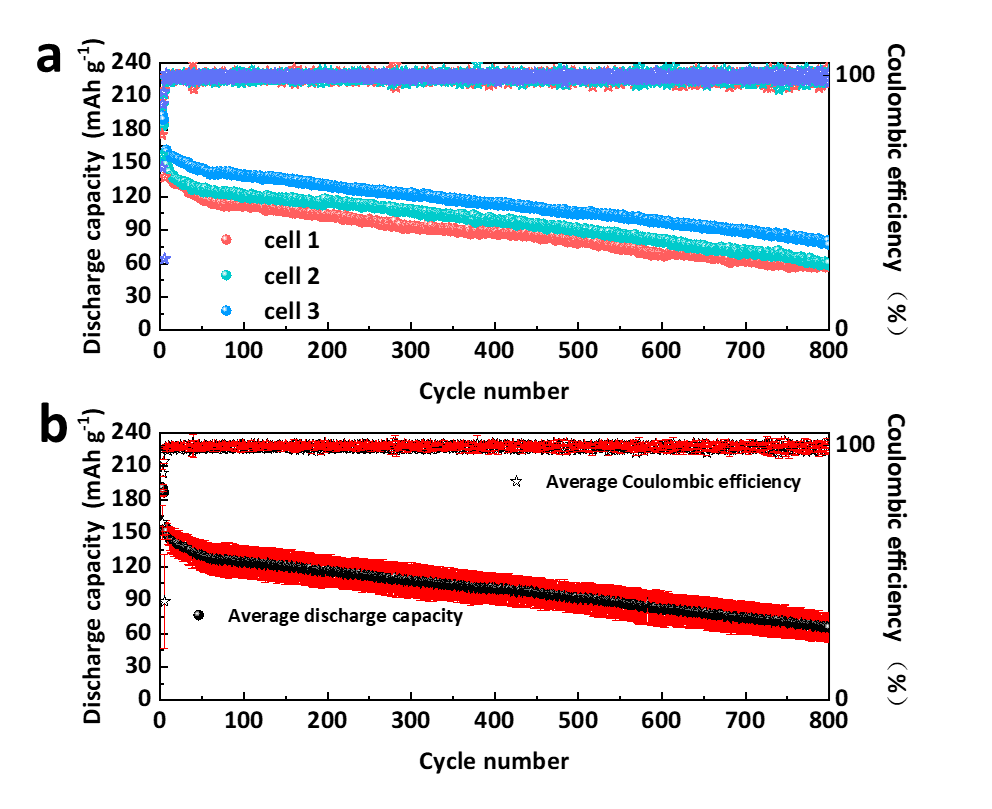


**Figure S9.** a) Cycling performance of three Li/NCM811 cells under the same conditions with customized electrolyte and b) their average data at 10C rate.

**Characterization of the electrolyte-electrode interphase**


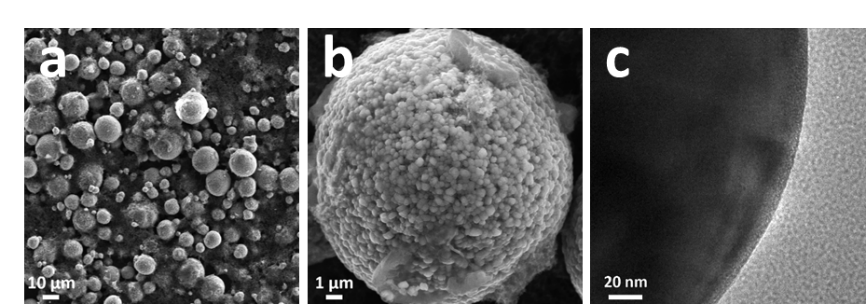


**Figure S10.** a,b) SEM and c) TEM images of uncycled NCM811 electrode.

**Scheme S1** Possible reaction mechanisms of FEC decomposition involved in LiF generation.


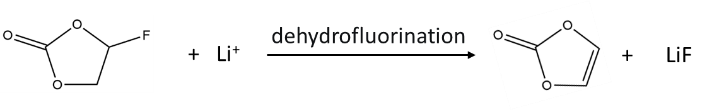


**Scheme S2** Possible reaction mechanisms of TPBX decomposition.


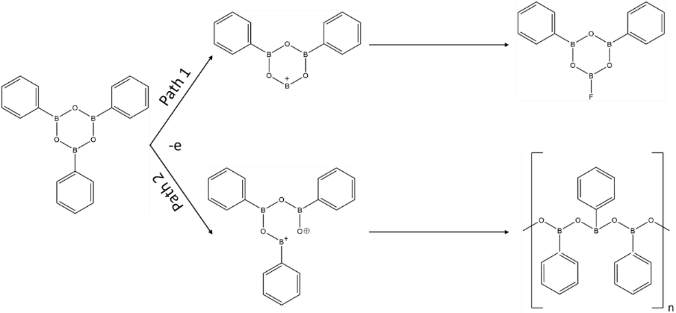


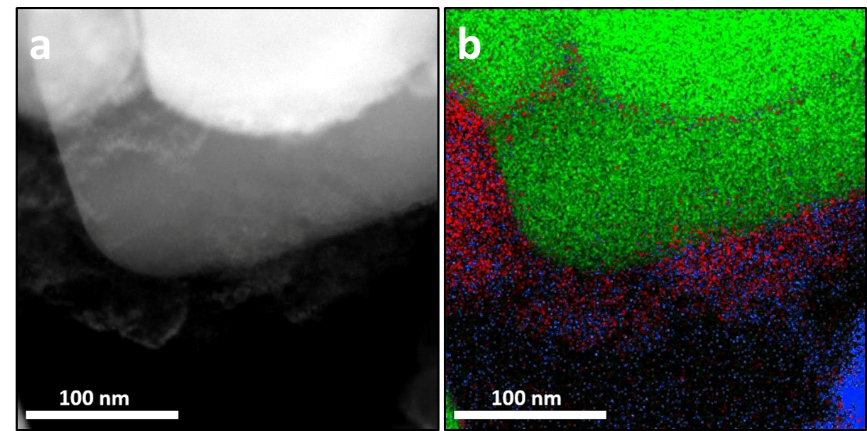


**Figure S11.** FIB-TEM images of NCM811 electrodes in customized electrolyte after 800 cycles at 10C rate.


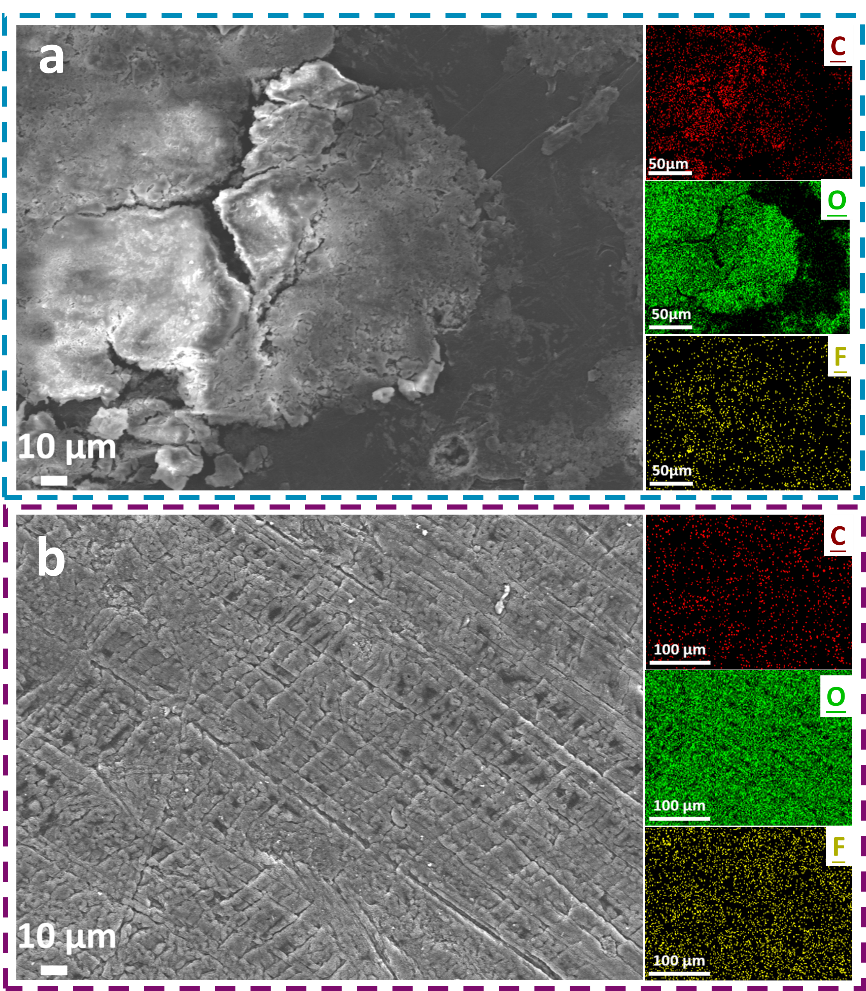


**Figure S12.** EDS mapping of lithium anode electrode surface after 800 cycles at 10C in (a) blank electrolyte and (b) customized electrolyte.


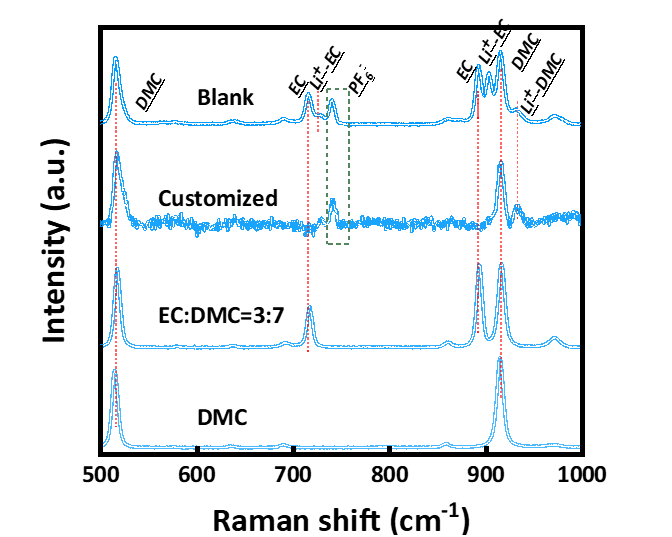


**Figure S13.** Raman spectra of blank electrolyte, customized electrolyte, mixed solvents of EC with DMC, and DMC solvent.

**The self-discharge tests**


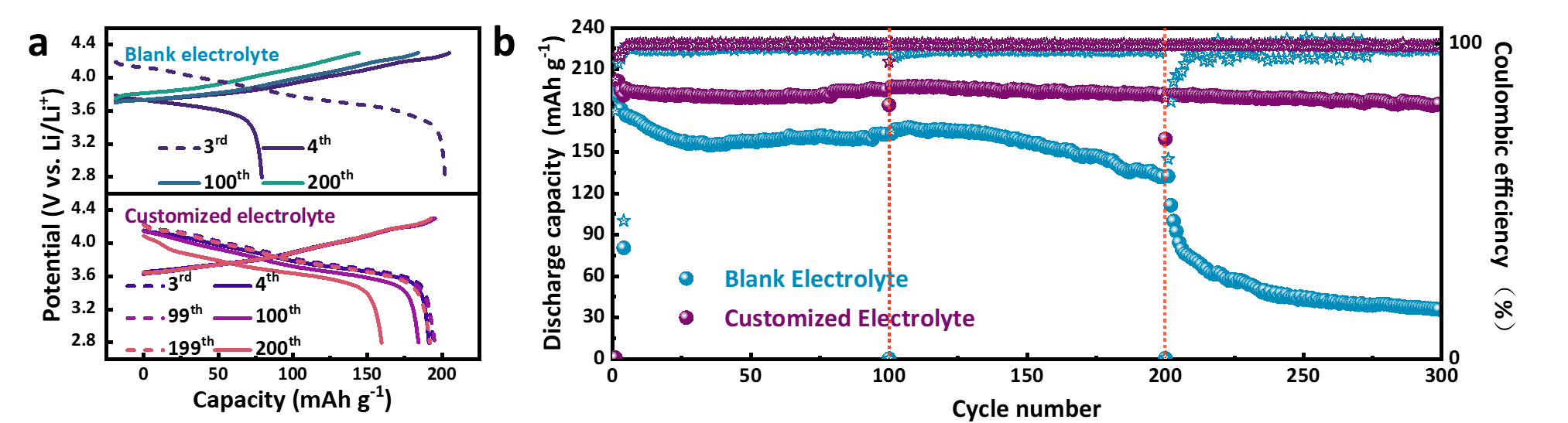


**Figure S14.** a) Corresponding first charge and discharge curves of Li/NCM811 cells after standing for 7 days. b) Cyclic performance of NCM811 electrodes after standing for 7 days at specific cycle numbers.

**The higher cutoff-voltage test**


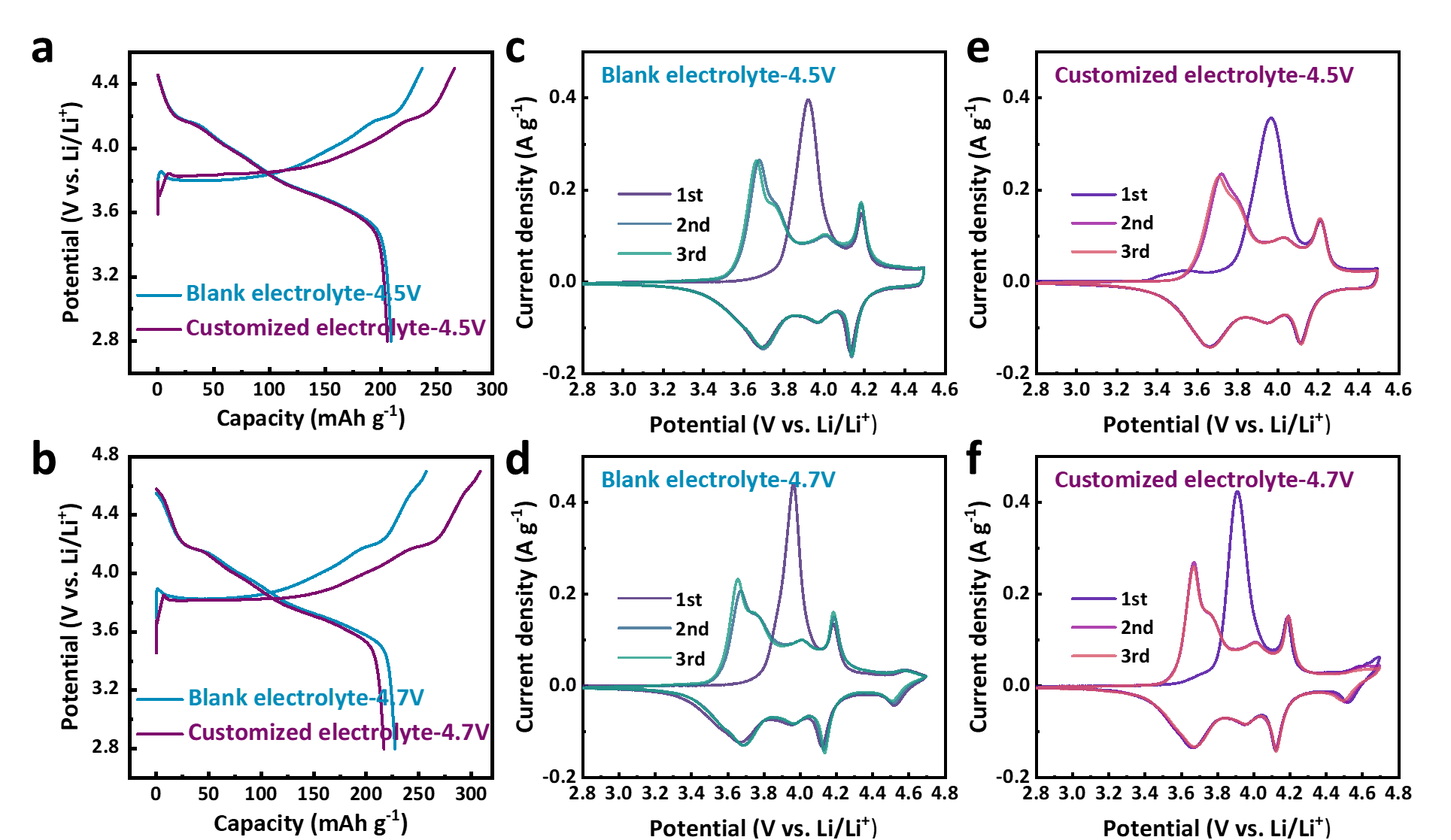


**Figure S15.** Charge-discharge curves of NCM811 electrodes in two electrolytes at first cycle with the charge cutoff voltage of a) 4.5V and b) 4.7V. CV curves of NCM811 electrodes in blank electrolyte with a charge cut-off voltage of c) 4.5V and d) 4.7V. CV curves of NCM811 electrodes in customized electrolyte with a charge cut-off voltage of e) 4.5V and f) 4.7V.

**The pouch cell tests**


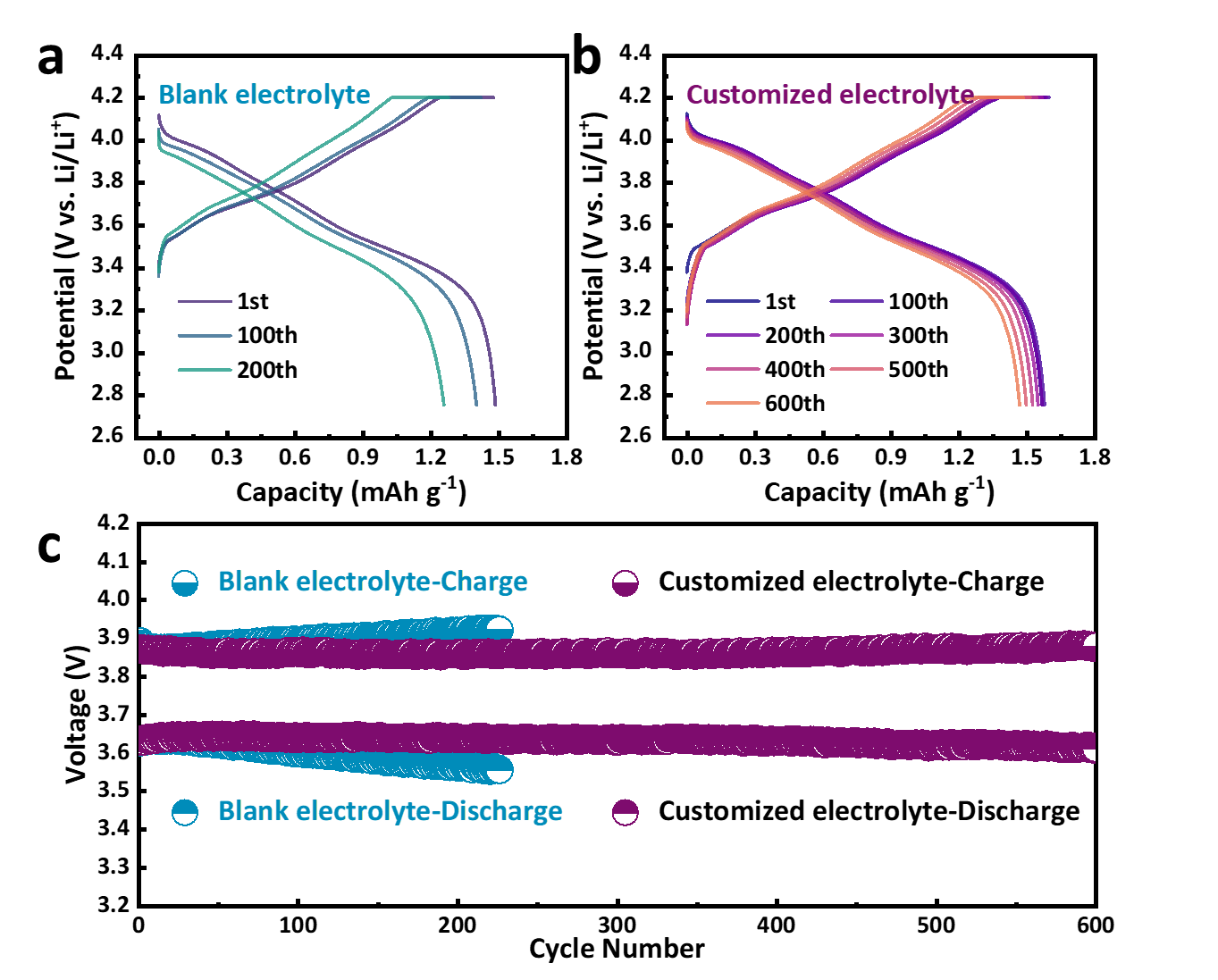


**Figure S16.** Charge-discharge curves of Graphite/NCM811 pouch cells with a) blank electrolyte and b) customized electrolyte at specific cycles. c) Charge-discharge medium voltage of Graphite/NCM811 pouch cells during cycling.

From Figure S16a and 16b that the charging capacity of the pouch cells consists of two parts: constant current charging and constant voltage charging. After using the customized electrolyte, the ratio of constant current charging capacity increases while that of constant voltage charging capacity decreases, indicating that the polarization of the cell decreases. This is also confirmed by the charge and discharge medium voltage of the pouch cells in Figure S16c. The charge and discharge medium voltage difference of the pouch cell using blank electrolyte increases continuously from the beginning of the cycle, while the charge and discharge medium voltage difference of the pouch cell using customized electrolyte has little change, which represents that less energy is consumed during charging and more energy is released during discharging to achieve higher energy conversion.


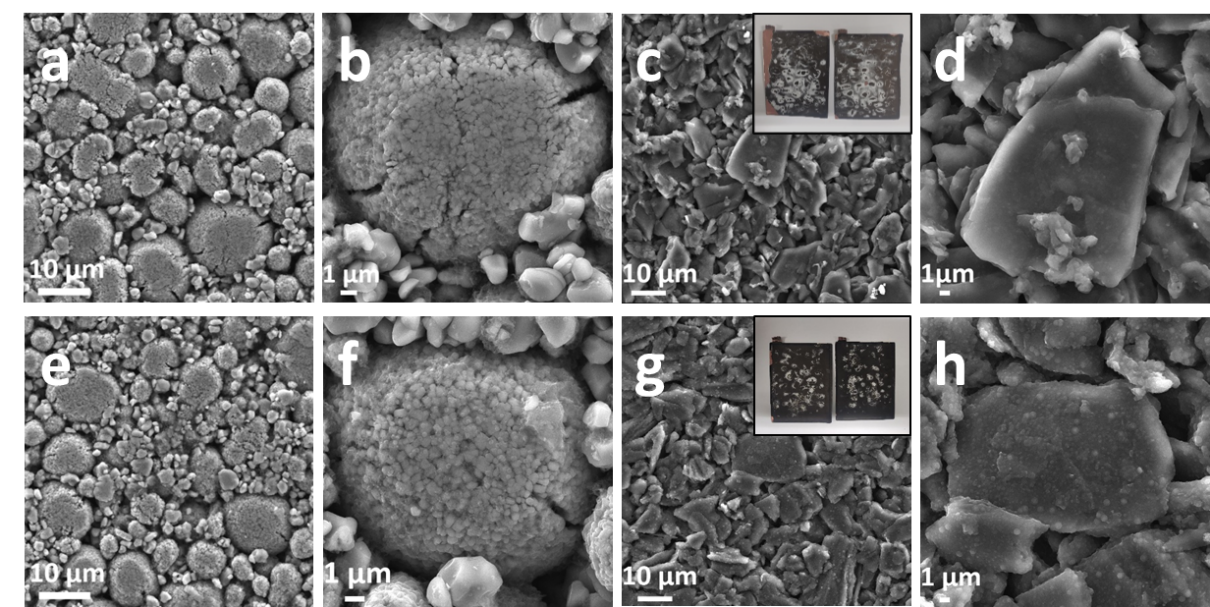


**Figure S17.** SEM images of cycled a,b) NCM811 electrodes and c,d) graphite electrodes in pouch cells with blank electrolyte. SEM images of cycled e,f) NCM811 electrodes and g,h) graphite electrodes in pouch cells with customized electrolyte.

The pouch cells are disassembled and the electrode are characterized by SEM. The cracks of secondary particles on the cathode electrodes and fragments on the anode electrode particles in customized electrolyte are less than those with blank electrolyte. That is to say, compared with the electrode in blank electrolyte, the electrode in customized electrolyte has undergone more cycles but less deterioration. This further shows that customized electrolyte can protect electrode materials to reduce the degree of breakage and thus improve the cell cycle life under practical application conditions.

**Table S1.** Detailed formulations of different electrolytes.

| Electrolyte | Lithium salt (LiPF_6_) | Solvent | Additives |
| --- | --- | --- | --- |
| 1# | 1M | DMC | / |
| 2# | 1.5M | DMC | / |
| 3# | 2M | DMC | / |
| 4# | 1.5M | DMC | 4 vol% VC |
| 5# | 1.5M | DMC | 4 vol% VC  + 1 wt% TPBX |
| Customized electrolyte | 1.5M | DMC | 4 vol% VC+ 1 wt% TPBX  + 6 vol%FEC |

**MD calculation methods**

1. **Simulation details**

Classical molecular dynamics (MD) simulations on two electrolyte systems, i.e., 1 M LiPF_6_ dissolved in the mixture of ethylene carbonate (EC) and dimethyl carbonate (DMC) in the blank system, or 1.5 M LiPF_6_ dissolved in the mixture of fluoroethylene carbonate (FEC), vinylene carbonate (VC), and DMC in the customized system, was performed using GROMACS^[1]^ program package. Specifically, in the MD simulations, the blank system consists of 100 LiPF_6_, 450 EC, and 796 DMC, while the customized system consists of 100 LiPF_6_, 44 VC, 55 FEC, and 758 DMC, respectively. An all-atom, all-flexible non-polarizable force field was adopted in this study. Detailed force field parameters, mostly taken from the OPLS^[2]^ force field, with the parameters that are missing in OPLS taken from GAFF,^[3]^ are summarized in the Supporting Information. The partial charges were fitted by Restrained electrostatic potential (RESP) method.^[4]^ A charge scaling scheme was adopted to account for the electronic polarization effect in an average manner, with charges of all atoms were scaled by $\frac{1}{\sqrt{2}}$, which is close to the optical dielectric constant of the solvents.^[5]^ A cut-off distance of 1.2 Å was adopted for both the real-space electrostatic interaction and the van der Waals interaction described by Lennard-Jones potential. The electrostatic interaction in the reciprocal space was handled by the smooth particle mesh ewald (SPME) method.^[6]^ Both systems were firstly equilibrated by isobaric-isothermal (NPT) simulation, coupled to a Parrinello-Rahman barostat^[7]^ of pressure $P=$ 0.1 MPa and a Nosé-Hoover thermostat^[8]^ with temperature gradually reduced from 2000 K to 298.15 K in 200 ns. Then the systems were further equilibrated by NPT simulation with $P=$ 0.1 MPa and $T=$ 298.15 K for 100 ns. Cubic periodic boundary condition (PBC) was applied to the electrolyte systems, and the average length of PBC cell for the blank and customized system is 49.70 Å and 56.59 Å, corresponding to the mass density of 1.16 g/cm^3^ and 1.26 g/cm^3^, respectively. Then the simulated mass density are in reasonable agreement with the experimental measurement of 1.16 g/cm^3^.and 1.26 g/cm^3^, the PBC boxes of the systems were adjusted to match the experimental densities for the MD with canonical (NVT) ensemble, coupled to a Nosé-Hoover thermostat,^[8]^ for 200 ns with $T=$ 298.15 K. The positions of the system were stored every 50 fs, and then analyzed by in-house codes, as detailed below.

1. **Structural analyses**
   1. *Radial distribution function and cumulative distribution function*

The radial distribution function (RDF) and the corresponding cumulative distribution function (CDF) are given by

$$\begin{aligned} g\left( r_{ij} \right)\text{ = }\frac{\left\langle\sum_{ij} \delta\left( r_{ij}-r \right) \right\rangle}{\rho_{j}4\pi r^{2}dr}\#\left( 1 \right) \end{aligned}$$

$$\begin{aligned} n\left( \text{r}_{ij} \right)=\rho_{j}\int_{0}^{\text{r}_{ij}} 4\pi r^{2}g\left( r \right)dr\#\left( 2 \right) \end{aligned}$$

in which $\text{r}_{ij}$ is the distance between species $i$ and $j$ and $\rho_{j}$ is the average number density of $j$. In the numerator of Equation (1), $\left\langle\right\rangle$ denotes the ensemble average and $\delta\left( r-r_{ij} \right)$ denotes Kronecker delta symbol, which is 1 if $r_{ij}\in[r,r+dr)$ and is 0 otherwise. Ideally, $dr\to0$, while in the numerical analyses, we adopted $dr=$ 0.01 Å for calculating Equation (1) and (2).

- 1. *Iso-density distribution*

RDF in Equation (1) is isotropic in the sense that it depends only on the distance between species $i$ and $j$. Such treatment is reasonable because liquid is isotropic in space for the thermal translational and rotational motions of the molecules that are not restricted around their potential minima in the system. Nonetheless, since a molecule generally owns its specific shape, which is generally not ball-like except for a single atom molecule/ion such as Li^+^, the actually local distribution around a solvent molecule is anisotropic in space. In order to illustrate such three dimensionally anisotropic distribution, we transform to molecular frame to observe the iso-density distribution of ions around the solvent molecule. Specifically, we took the relatively “rigid” part of the solvent molecule, i.e., the noncollinear –F-P-F– that is nearly flat, to define the molecular frame, and transformed the spatial distribution around each solvent molecule from the lab frame to the molecular frame. By such treatment, the spatial distribution is observed in the same molecular frame, and the iso-density distribution of selected number density was plotted because a full representation takes four dimensions. The average number densities of each species are $\rho_{{Li}^{+}}=0.0006 Å^{-3}$in the blank system, as well as $\rho_{{Li}^{+}}=0.0006 Å^{-3}$in customized system, respectively. The average number densities of the respective ions are $\rho_{{Li}^{+}}=0.00055 Å^{-3}$in the blank system, and are $\rho_{{Li}^{+}}=0.00081 Å^{-3}$, and in the customized system.

- 1. *Procedure for identifying -anion aggregates*

Beginning with a lithium in a frame, we search for anions within a specified cutoff. If all the anions within the cutoff are discovered, we then search for $\mathrm{Li}^{+}$, other than the initial one, within the same distance cutoff but now centered on the anions found in the previous step. We continue this cyclic search until no new ions can be found, and we identify the aggregate composition by counting the $\mathrm{Li}^{+}$’s and anions. Double counting is avoided, and a cutoff of 5.1Å, which corresponds to the first minimum of $g\left( r_{\mathrm{Li}^{+}-PF_{6}^{-}}^{\mathrm{cm}} \right)$ is utilized in this study. The operations are then repeated from a different starting $\mathrm{Li}^{+}$. When all $\mathrm{Li}^{+}$’s and anions in the current frame have been assigned to a certain aggregate, we move on to the next frame.

1. **Force Field Parameters**

We employed classical molecular dynamics (MD) simulations to study two electrolyte systems using an all-atom, all-flexible model with non-polarizable force field in the following form,

$$V=\sum_{bonds} k_{b}\left( r_{ij}-r_{0} \right)^{2}+\sum_{angles} k_{\theta}\left( \theta_{jik}-\theta_{0} \right)^{2}$$

$$+\sum_{dihedrals} \frac{1}{2}\left\{ V_{1}\left[ 1+\cos\left( \varphi\right) \right]+V_{2}\left[ 1-\cos\left( 2\varphi\right) \right]+V_{3}\left[ 1+\cos\left( 3\varphi\right) \right] \right\}$$

$$\begin{aligned} +\sum_{i} \sum_{j>i} \left\{ 4\epsilon_{ij}\left[ \left( \frac{\sigma_{ij}}{r_{ij}} \right)^{12}-\left( \frac{\sigma_{ij}}{r_{ij}} \right)^{6} \right]+\frac{1}{4\pi\varepsilon_{0}}\frac{q_{i}q_{j}}{r_{ij}} \right\}\# \end{aligned}$$

in which the first three terms represent the bond, angle and dihedral interactions, and the last term represents the long-range interactions, including van der Waals and Coulombic interactions. Anion and solvent molecules in two electrolyte systems, along with their designated atomic types, are depicted in Figure S18. All the force field parameters, mostly taken from the OPLS force field, with the parameters that are missing in OPLS taken from GAFF, are summarized in Supplementary Table 2 to 6.

| 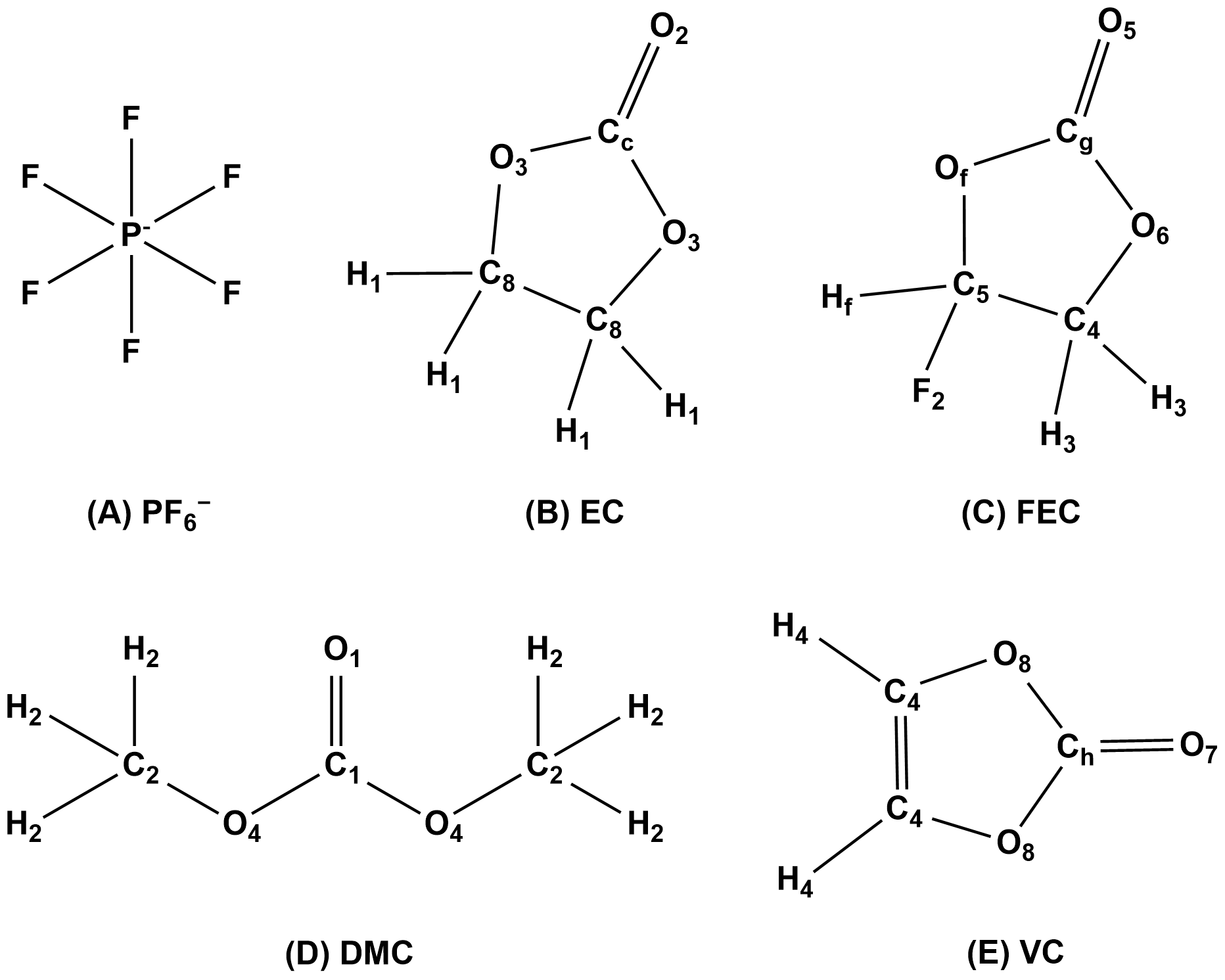 |
| --- |

**Figure S18.** All-atom representation of anion and solvent molecules.

**Table** **S2**. Bond parameters ($k_{b}$ in kcal·mol^-1^·Å^-2^ and $r_{0}$ in Å).

| PF_6_ | | | | | EC | | | | | | | | | | |
| --- | --- | --- | --- | --- | --- | --- | --- | --- | --- | --- | --- | --- | --- | --- | --- |
| Bonds | *k_b_* | | *r_0_* | | Bonds | | *k_b_* | | *r_0_* | | Bonds | | *k_b_* | | *r_0_* |
| P-F | 370.459 | | 1.606 | | Cc-O2 | | 570.0 | | 1.229 | | C8-C8 | | 310.0 | | 1.526 |
| FEC | | | | | C8-H1 | | 340.0 | | 1.090 | | C8-O3 | | 320.0 | | 1.410 |
| Bonds | *k_b_* | | *r_0_* | | Cc-O3 | | ^c^377.5 | | ^b^1.369 | |  | |  | |  |
| C5-O6 | 320.0 | | 1.410 | | DMC | | | | | | | | | | |
| C5-H3 | 340.0 | | 1.090 | | Bonds | | *k_b_* | | *r_0_* | | Bonds | | *k_b_* | | *r_0_* |
| C4-Hf | 340.0 | | 1.090 | | C1-O1 | | 570.0 | | 1.229 | | C2-H2 | | 340.0 | | 1.090 |
| Cg-O5 | 570.0 | | 1.229 | | C1-O4 | | ^c^408.8 | | ^b^1.345 | | C2-O4 | | 320.0 | | 1.410 |
| C4-F2 | 367.0 | | 1.380 | | VC | | | | | | | | | | |
| C4-Of | 320.0 | | 1.410 | | Bonds | | *k_b_* | | *r_0_* | | Bonds | | *k_b_* | | *r_0_* |
| C4-C5 | 310.0 | | 1.526 | | Ch-O8 | | ^c^377.5 | | ^b^1.369 | | C9-C9 | | 549.0 | | 1.350 |
| Cg-O6 | ^c^377.5 | | ^b^1.369 | | C9-O8 | | ^c^358.3 | | ^b^1.385 | | Ch-O7 | | 570.0 | | 1.229 |
| Cg-Of | ^c^377.5 | ^b^1.369 | | C9-H4 | | ^c^359.0 | | ^b^1.077 | |  | |  | |  | |

^a^Parameters without explicit citation were taken from AMBER force field for PF_6_ , EC, FEC, DMC and VC; ^b^Parameters were taken from：ab initio calculations (MP2/6-31G*); ^c^Parameters were taken from GAFF.

**Table** **S3**. Angle parameters ($k_{\theta}$ in kcal·mol^-1^·radian^-2^ and $\theta_{0}$ in degree).

| PF_6_ | | | EC | | | | | |
| --- | --- | --- | --- | --- | --- | --- | --- | --- |
| Angles | *k_θ_* | *θ_0_* | Angles | *k_θ_* | *θ_0_* | Angles | *k_θ_* | *θ_0_* |
| F-P-F | 139.22 | 90.0 | C8-C8-H1 | 50.0 | 109.5 | O3-Cc-O3 | ^c^75.4 | ^b^110.1 |
| FEC | | | H1-C8-H1 | 35.0 | 109.5 | C8-O3-Cc | ^c^65.8 | ^b^108.4 |
| Angles | *k_θ_* | *θ_0_* | C8-C8-O3 | 50.0 | 109.5 | O3-Cc-O2 | ^c^74.2 | ^b^125.0 |
| F2-C4-Of | ^c^71.0 | ^b^109.4 | H1-C8-O3 | 50.0 | 109.5 |  |  |  |
| F2-C4-C5 | ^c^65.7 | ^b^110.0 |  |  |  |  |  |  |
| C5-O6-Cg | ^c^65.8 | ^b^108.4 | DMC | | | | | |
| O6-Cg-Of | ^c^75.4 | ^b^110.1 | Angles | *k_θ_* | *θ_0_* | Angles | *k_θ_* | *θ_0_* |
| O6-Cg-O5 | ^c^74.2 | ^b^125.0 | O1-C1-O4 | ^c^74.6 | ^b^126.3 | H2-C2-H2 | 35.0 | 109.5 |
| C4-Of-Cg | ^c^65.6 | ^b^108.9^b^ | O4-C1-O4 | ^c^77.7 | ^b^107.5 | H2-C2-O4 | 50.0 | 109.5 |
| Of-Cg-O5 | ^c^74.2 | ^b^125.0 | C2-O4-C1 | ^c^64.9 | ^b^113.3 |  |  |  |
| Hf-C4-Of | 50.0 | 109.5 |  |  |  |  |  |  |
| F2-C4-Hf | 35.0 | 109.5 | VC | | | | | |
| H3-C5-H3 | 35.0 | 109.5 | Angles | *k_θ_* | *θ_0_* | Angles | *k_θ_* | *θ_0_* |
| C4-C5-O6 | 50.0 | 109.5 | O8-Ch-O7 | ^c^73.9 | ^b^ 126.0 | C9-C9-O8 | ^c^73.7 | ^b^108.7 |
| C5-C4-Of | 50.0 | 109.5 | O8-Ch-O8 | ^c^76.2 | ^b^107.9 | H4-C9-O8 | ^c^50.7 | ^b^117.3 |
| C4-C5-H3 | 50.0 | 109.5 | C9-C9-H4 | ^c^46.9 | ^b^134.0 | C9-O8-Ch | ^c^66.7 | ^b^107.3 |
| C5-C4-Hf | 50.0 | 109.5 |  |  |  |  |  |  |
| H3-C5-O6 | 50.0 | 109.5 |  |  |  |  |  |  |

^a^Parameters without explicit citation were taken from AMBER force field for PF_6_ , EC, FEC, DMC and VC; ^b^Parameters were taken from：ab initio calculations (MP2/6-31G*); ^c^Parameters were taken from GAFF.

**Table** **S4**. Dihedral parameters (*V_i_* in kcal·mol^-1^).

| EC | | | | | | | |
| --- | --- | --- | --- | --- | --- | --- | --- |
| *dihedral* | *V1* | *V2* | *V3* | *dihedral* | *V1* | *V2* | *V3* |
| ^d^C8-O3-Cc-O2 | -0.574 | -0.997 | 0 | ^d^H1-C8-O3-Cc | 0 | 0 | 0.760 |
| ^d^O3-C8-C8-H1 | 0 | 0 | 0 | ^d^C8-C8-O3-Cc | 0.650 | -0.250 | 0.670 |
| ^d^H1-C8-C8-H1 | 0 | 0 | 0.360 | ^d^C8-O3-Cc-O3 | -0.574 | -0.997 | 0 |
| ^d^O3-C8-C8-O | 0 | 0 | 0.288 |  |  |  |  |
| FEC | | | | | | | |
| *dihedral* | *V1* | *V2* | *V3* | *dihedral* | *V1* | *V2* | *V3* |
| ^d^H3-C5-O6-Cg | 0 | 0 | 0.760 | ^d^C4-C5-O6-Cg | 0.650 | -0.250 | 0.670 |
| ^d^C5-O6-Cg-Of | -0.574 | -0.997 | 0 | ^d^C4-Of-Cg-O6 | -0.574 | -0.997 | 0 |
| ^d^C5-O6-Cg-O5 | -0.574 | -0.997 | 0 | F2-C4-Of-Cg | 0 | 0 | 2.300 |
| ^d^Of-C4-C5-H3 | 0 | 0 | 0 | Of-C4-C5-O6 | 0 | 2.00 | 0.288 |
| ^d^Hf-C4-C5-H3 | 0 | 0 | 0.360 | O6-C5-C4-F2 | 0 | 0 | 2.800 |
| ^d^Hf-C4-Of-Cg | 0 | 0 | 0.760 | F2-C4-C5-H3 | 0 | 0 | 2.800 |
| ^d^C5-C4-Of-Cg | 0.650 | -0.250 | 0.670 | O6-C5-C4-Hf | 0 | 0 | 2.800 |
| DMC | | | | | | | |
| *dihedral* | *V1* | *V2* | *V3* | *dihedral* | *V1* | *V2* | *V3* |
| C2-O4-C1-O1 | -0.574 | -0.997 | 0 | C2-O4-C1-O4 | -0.574 | -0.997 | 0 |
| H2-C2-O4-C1 | 0 | 0 | 0.760 |  |  |  |  |
| VC | | | | | | | |
| *dihedral* | *V1* | *V2* | *V3* | *dihedral* | *V1* | *V2* | *V3* |
| ^d^C9-C9-O8-Ch | 0.650 | -0.250 | 0.670 | O8-C9-C9-O8 | 0 | 53.20 | 0 |
| ^d^C9-O8-Ch-O8 | -0.574 | -0.997 | 0 | O8-C9-C9-H4 | 0 | 53.20 | 0 |
| ^d^C9-O8-Ch-O7 | -0.574 | -0.997 | 0 | H4-C9-C9-H4 | 0 | 53.20 | 0 |
| ^d^H4-C9-O8-Ch | 0 | 0 | 0.760 |  |  |  |  |

^a^Parameters without explicit citation were taken from AMBER force field for EC, FEC, DMC and VC; ^d^Parameters were taken from OPLS force field.

.

**Table** **S5**. Improper dihedral parameters (*V_i_* in kcal·mol^-1^).

| EC | | | |
| --- | --- | --- | --- |
| Improper dihedral | V1 | V2 | V3 |
| O2-Cc-O3-O3 | 0 | 21.0 | 0 |
| FEC | | | |
| Improper dihedral | V1 | V2 | V3 |
| O5-Cg-Of-O6 | 0 | 21.0 | 0 |
| DMC | | | |
| Improper dihedral | V1 | V2 | V3 |
| O1-C1-O4-O4 | 0 | 21.0 | 0 |
| VC | | | |
| Improper dihedral | V1 | V2 | V3 |
| O7-Ch-O8-O8 | 0 | 21.0 | 0 |
| O8-C9-H4-C9 | 0 | 21.0 | 0 |

^a^Parameters without explicit citation were taken from AMBER force field for EC, FEC, DMC and VC.

**Table** **S6**. Lennard-Jones parameters with the atomic partial charges scaled by a factor of $\frac{1}{\sqrt{2}}$ ($q$ in elementary electron unit $e$, $\sigma_{i}$ in Å and $\epsilon_{i}$ in kcal·mol^-1^)*^a^*

| EC | | | | | PF_6_ | | | | |
| --- | --- | --- | --- | --- | --- | --- | --- | --- | --- |
| Types | Atom | $q$ | $\sigma_{i}$ | $\epsilon_{i}$ | Types | Atom | $q$ | $\sigma_{i}$ | $\epsilon_{i}$ |
| CT | C8 | 0.140 | 3.500 | 0.066 | P | P | 1.340 | 3.740 | 0.200 |
| C | Cc | 0.470 | 3.750 | 0.105 | F | F | -0.39 | 3.1181 | 0.061 |
| OS | O3 | -0.192 | 2.900 | 0.140 | DMC | | | | |
| O | O2 | -0.486 | 2.960 | 0.210 | Types | Atom | $q$ | $\sigma_{i}$ | $\epsilon_{i}$ |
| H1 | H1 | 0.030 | 2.500 | 0.030 | C | C1 | 0.470 | 3.750 | 0.105 |
| FEC | | | | | O | O1 | -0.470 | 2.960 | 0.210 |
| Types | Atom | $q$ | $\sigma_{i}$ | $\epsilon_{i}$ | OS | O4 | -0.248 | 2.900 | 0.140 |
| CT | C5 | 0.140 | 3.500 | 0.066 | CT | C2 | 0.140 | 3.500 | 0.066 |
| C | Cg | 0.470 | 3.750 | 0.105 | H1 | H2 | 0.036 | 2.500 | 0.030 |
| O | O5 | -0.470 | 2.960 | 0.210 | VC | | | | |
| OS | Of | -0.424 | 2.900 | 0.140 | Types | Atom | $q$ | $\sigma_{i}$ | $\epsilon_{i}$ |
| H1 | Hf | 0.074 | 2.500 | 0.030 | C | Ch | 0.470 | 3.750 | 0.105 |
| OS | O6 | -0.205 | 2.900 | 0.140 | CM | C9 | 0.088 | 3.550 | 0.076 |
| H1 | H3 | 0.040 | 2.500 | 0.030 | O | O7 | -0.414 | 2.960 | 0.210 |
| F | F2 | -0.242 | ^a^1.750 | ^a^0.061 | OS | O8 | -0.214 | 2.900 | 0.140 |
| CT | C4 | 0.392 | ^a^1.908 | ^a^0.109 | H1 | H4 | 0.098 | 2.500 | 0.030 |

^d^Parameters without explicit citation were taken from OPLS force field for PF_6,_ EC, FEC, DMC and VC; ^a^Parameters were taken from AMBER force field. The Lennard-Jones parameters between different atomic types *i* and *j*, $\epsilon_{ij}$ and $\sigma_{ij}$, are handled with Lorentz-Berthelot mixing rule, i.e., $\epsilon_{ij}=\sqrt{\epsilon_{i}\epsilon_{j}}$ and $\sigma_{ij}=(\sigma_{i}+\sigma_{j})/2$.

**Supporting Reference**

[1] M. J. Abraham, T. Murtola, R. Schulz, S. Páll, J. C. Smith, B. Hess, E. Lindahl, *SoftwareX* **2015**, *1-2*, 19-25.

[2] W. L. Jorgensen, D. S. Maxwell, J. Tirado-Rives, *J. Am. Chem. Soc.* **1996**, *118*, 11225-11236.

[3] J. Wang, R. M. Wolf, J. W. Caldwell, P. A. Kollman, D. A. Case, *J. Comput. Chem.* **2004**, *25*, 1157-1174.

[4] C. I. Bayly, P. Cieplak, W. Cornell, P. A. Kollman, *J. Phys. Chem.* **2002**, *97*, 10269-10280.

[5] J. Gu, Y. Jia, X. Ren, S. Li, T. Yan, *J. Mol. Liq.* **2023**, *369*, 120815.

[6] U. Essmann, L. Perera, M. L. Berkowitz, T. Darden, H. Lee, L. G. Pedersen, *J. Chem. Phys.* **1995**, *103*, 8577-8593.

[7] M. Parrinello, A. Rahman, *J. Appl. Phys.* **1981**, *52*, 7182-7190.

[8] S. Nosé, *J. Chem. Phys.* **1984**, *81*, 511-519.
